# Modeling the Interruption of the Transmission of Soil-Transmitted Helminths Infections in Kenya: Modeling Deworming, Water, and Sanitation Impacts

**DOI:** 10.3389/fpubh.2021.637866

**Published:** 2021-03-24

**Authors:** Collins Okoyo, Graham Medley, Charles Mwandawiro, Nelson Onyango

**Affiliations:** ^1^Eastern and Southern Africa Centre of International Parasite Control, Kenya Medical Research Institute, Nairobi, Kenya; ^2^School of Mathematics, University of Nairobi, Nairobi, Kenya; ^3^Faculty of Public Health and Policy, London School of Hygiene and Tropical Medicine, London, United Kingdom

**Keywords:** mathematical modeling, deworming, soil-transmitted helminths, water sanitation and hygiene, Kenya

## Abstract

Kenya, just like other countries with endemic soil-transmitted helminths (STH), has conducted regular mass drug administration (MDA) program for the last 5 years among school aged children as a way to reduce STH infections burden in the country. However, the point of interruption of transmission of these infections still remains unclear. In this study, we developed and analyzed an age structured mathematical model to predict the elimination period (i.e., time taken to interrupt STH transmission) of these infections in Kenya. The study utilized a deterministic age structured model of the STH population dynamics under a regular treatment program. The model was applied to three main age groups: pre-school age children (2–4 years), school age children (5–14 years), and adult populations (≥15 years) and compared the impact of two interventions on worm burden and elimination period. The model-simulated results were compared with the 5 year field data from the Kenyan deworming program for all the three types of STH (*Ascaris lumbricoides, Trichuris trichiura*, and hookworm). The model demonstrated that the reduction of worm burden and elimination period depended heavily on four parameter groups; drug efficacy, number of treatment rounds, MDA and water, sanitation and hygiene (WASH) coverage. The analysis showed that for STH infections to be eliminated using MDA alone in a short time period, 3-monthly MDA plan is desired. However, complementation of MDA with WASH at an optimal (95%) coverage level was most effective. These results are important to the Kenyan STH control program as it will guide the recently launched *Breaking Transmission Strategy*.

## 1. Introduction

Soil-transmitted helminths (STH) are part of a group of diseases categorized by the World Health Organization (WHO) as neglected tropical diseases (NTDs). The three main types of STH are *Ascaris lumbricoides* (roundworm), *Trichuris trichiura* (whipworm), and hookworms (*Necator americanus* and *Ancylostoma duodenale*) (1, 2). STH are estimated to be endemic in about 166 countries and affect more than two billion people globally (1). A further four billion people are estimated to be at risk. These infections occur mainly in the rural areas of Sub-Saharan Africa, Latin America, China, and South East Asia (3).

STH are mainly caused by lack of safe drinking water, proper sanitation (practice of open defecation), and hygiene (poor practice of handwashing and walking barefoot on contaminated soil) (4). STH are generally transmitted through ingestion of nematode eggs from contaminated soil (*A. lumbricoides* and *T. trichiura*) or through active penetration of the skin by larvae in the soil (hookworm) (5). As such, the infection prevalence and intensity are strongly inversely correlated with the access to and use of improved sources of water, sanitation and hygiene (WASH) (6). Infected individuals show a broad range of symptoms including nausea, tiredness, abdominal pain, and loss of appetite that can attenuate malnutrition and increased rates of anemia (7).

STH can be effectively controlled through preventive chemotherapy (PC) to most at risk groups of individuals using any of the four WHO-recommended anthelminthic drugs; albendazole, mebendazole, levamisole, or pyrantel. However, albendazole and mebendazole are the most preferred drugs because of their higher efficacy against all the three geohelminths (8, 9). Further, WHO recommends that complimentary WASH interventions should be implemented to sustain the control achieved through PC (10). WASH interventions are vital in accelerating the interruption of STH transmission such that PC can be safely stopped in the long run without the risk of infection rebound (5, 11). However, it is unclear at what minimum coverage, effectiveness and time frame WASH should be implemented.

Majority of the STH endemic countries are now implementing mass drug administration (MDA) programs, either through school-based deworming (SBD) mainly targeting pre-school and school going children, or community-based deworming (CBD) targeting all age groups (12). CBD has been shown to be highly effective in reducing the community-wide morbidity associated with STH infections as compared to SBD programs, however it may be complex to implement unlike SBD programs which can be easily implemented through the school system (13). Although these programs have many benefits to the treated individuals, they do not prevent re-infections which can occur rapidly after treatment (14). Hence, there is need for individuals to adhere to treatment and programs to offer frequent and consistent MDA to maximize the benefits of PC (15). Both programs have two main advantages; reduction of worm burden, hence reduced morbidity for treated individuals, and reduction in further infection for all individuals (treated and untreated) as a result of overall reduction in transmission (16).

While researchers have disagreed over the years on the actual benefit of deworming on childrens' education outcomes, studies in which treatment is randomized at individual-level have shown to potentially underestimate the benefits of treatment and most often miss to capture externality benefits (17). However, randomization of treatment at school or community level allows for estimation of the overall program effect (17). Further, existing evidence still indicates that mass treatment of school children through MDA programs is a cost-effective health investment in low-resource settings (18).

Kenya has been implementing annual MDA through the SBD platform since the year 2012 targeting school going children (19–22). The aim of the Kenyan deworming program was to reduce the national STH infection burden to a level where the infections are no longer a public health problem [defined by a prevalence of below 1% (23)]. After 5 years (2012-2017) of implementation, the program has not achieved a community-wide treatment benefit due to four main challenges summarized as; (i) Which age group(s) should be targeted for treatment? pre-school aged children (PSAC), school aged children (SAC) or adults, (ii) How often should the treatment be delivered in each age group?, (iii) Under what conditions can transmission be eliminated by repeated treatment alone?, and (iv) Can elimination be achieved faster when contemporary interventions like WASH are added? Mathematical model would help us answer the above questions and inform on the right mix of strategies for the control of STH infections in Kenya.

In this paper we developed an age-structured mathematical model based on ordinary differential equations (ODE), which are part of deterministic models, to determine the impact of MDA and WASH interventions on worm burden and elimination period (i.e., time taken to interrupt STH transmission) specific to Kenyan infection transmission dynamics. Deterministic models have been widely applied to study STH transmission dynamics in various settings (11, 16, 24–32). We therefore used the modeling framework provided in these previous studies while incorporating additional important aspects to the model like full age-structure and comparison of impact of two interventions (MDA and WASH). We predicted the short-term (≤5 years) and long-term (>10 years) impact of the interventions under various MDA plans for each of the three STH species. The results of this model will be important to the Kenyan STH control program as well as to WHO since it clearly demonstrated the impact of the two key interventions on worm burden and elimination period. An important result at this time when control programs are rallying toward the WHO goal of STH elimination by the year 2030 (33).

## 2. Materials and Methods

### 2.1. Kenyan National Deworming Program Context

Kenya launched a country-wide national deworming program in the year 2012. The program aimed to deworm all school going children living in 66 sub-counties determined to be endemic of STH infections, and subsequently reduce the infection prevalence to below 1%. To enable targeted delivery of treatment and following WHO guidelines on STH control (13), school children were targeted for annual MDA. The process and impact of the program is continuously being evaluated through a robust monitoring and evaluation (M&E) program which is independently conducted by the Kenya Medical Research Institute (KEMRI) as described elsewhere (19). The results of the first 5-year (2012-2017) impact of the program is outlined elsewhere (20). The current modeling analysis therefore is aimed at addressing the challenges the program faced on the first 5-years of implementation resulting in its inability to achieve community-wide treatment benefit. For a detailed description of this program's design, impact and challenges, please see previous M&E reports (19–22). Specifically, we developed a full age-structured mathematical model to answer the earlier outlined questions and inform on the right mix of strategies for the control and elimination of STH infections in Kenya.

### 2.2. Model Conceptual Framework

The helminth lifecycle can be simplistically represented as population of mature worms in the human host (*M*_*i*_) and population of free-living infectious materials (i.e., eggs or larvae) in the environment (*L*). The host gets infected by getting into contact with the infectious materials in the environment and they also contaminate the environment with infectious materials. Unlike previous STH transmission models (16, 24, 26, 28), the current model stratified the host population into three commonly referred age groups: PSAC (2 to 4 years), SAC (5 to 14 years), and adults (above 14 years) (34, 35). Currently, there is increasing global interest in treating the PSAC and adults together with SAC, and studies have indicated that PSAC and adults if left untreated are likely to act as parasite reservoirs (36). It would therefore be important to understand their engagement in the community level transmission alongside the routinely treated SAC group.

**Figure 1:**
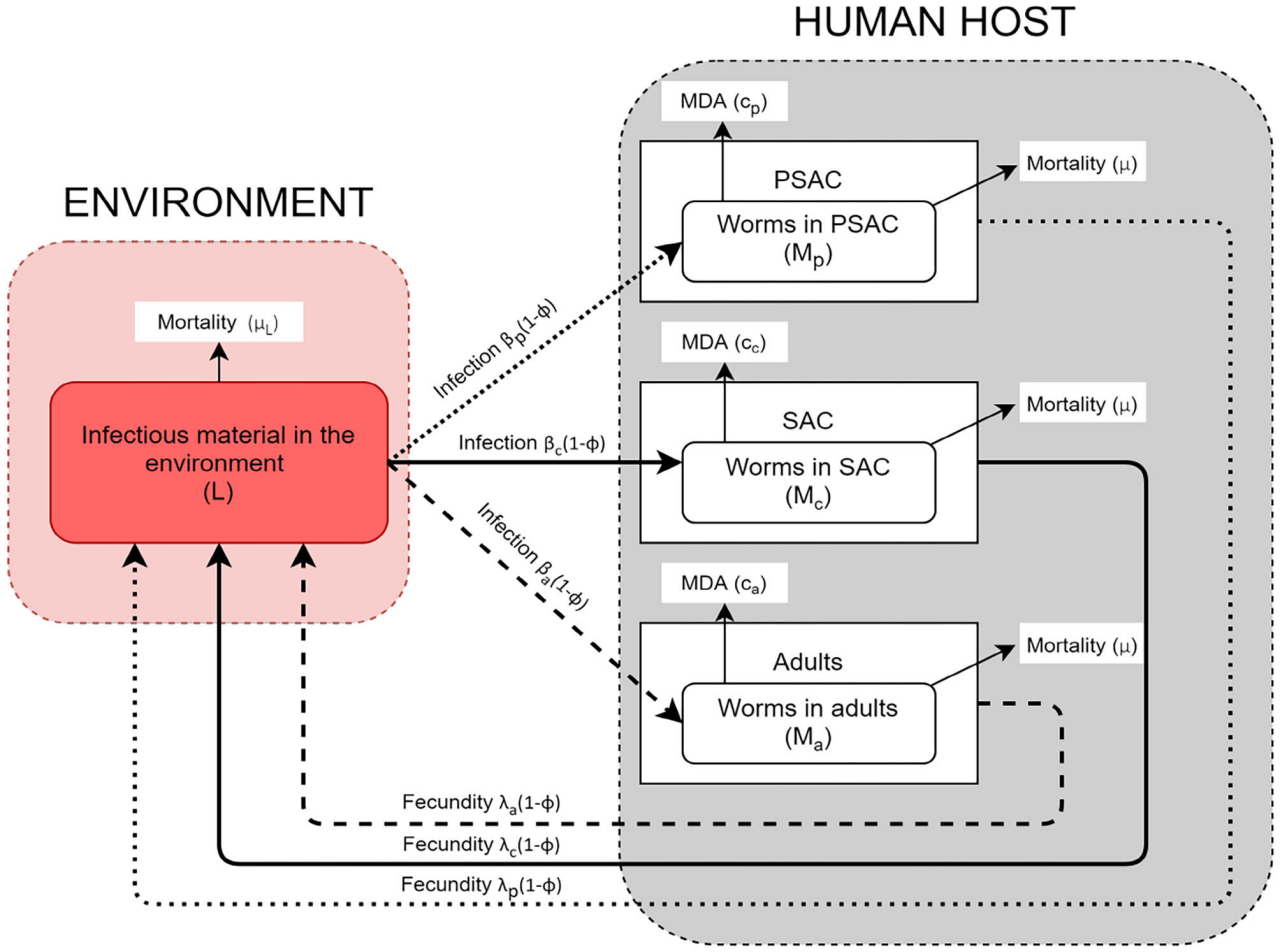
Conceptual framework showing a three age-structured model.

The model conceptual framework is given in Figure 1. In the framework, the three different host groups are assumed to acquire infections and consequently contaminate the environment at distinctively different rates; β_*i*_(1 − ϕ) and λ_*i*_(1 − ϕ), respectively. For each host, worm burden distribution is over-dispersed and assumed to follow negative-binomial distribution with aggregation parameter *k*, implying that majority of individual hosts harbor few worms while a minority of hosts have consistently higher worm burdens. The mature worms in each host group have similar mortality rate, μ, while the mortality rate for the free-living infectious materials in the environment is denoted by μ_*L*_. Additionally, the model incorporates two interventions, mass treatment (MDA) and WASH. The effect of mass treatment which is simulated as instantaneous killing of the mature worms in the host is given by the function *c*_*i*_. On the other hand, WASH which has a long term deterrent effect on the transmission and contamination of the environment with infectious materials, is simulated as the proportion of hosts in each group who have access to and use of improved water sources and sanitation facilities. It is important to note that here, we are simulating the combined effect of improved water source and sanitation, at a constant rate ϕ.

### 2.3. Model Specification

In this study, we formulated a three age-structured mathematical model for STH transmission based on the previous models by Chan et al. (16) and Truscott et al. (26). However, our model differs from these other models by explicitly including the dynamics of the infectious materials in the environment, complete categorization of the total population into three main age groups of interest to control programs, and comparison of the effect of two interventions (MDA and WASH). As explained in the conceptual framework, the human population was categorized into three age groups, namely; PSAC, SAC, and adults to enable us mimic a community-wide STH control program.We developed three model scenarios; first scenario is when no intervention was assumed (Model 1), second scenario is when only MDA intervention was assumed (Model 2), and last scenario is when both MDA and WASH interventions were assumed (Model 3).

#### 2.3.1. Model With No Intervention

The first model is when no intervention was assumed and it culminated into a four-dimensional system of ODE as follows.

(1) $$\begin{array}{l} \frac{dM_{p}}{dt}=\beta_{p}L-\mu M_{p}\\ \frac{dM_{c}}{dt}=\beta_{c}L-\mu M_{c}\\ \frac{dM_{a}}{dt}=\beta_{a}L-\mu M_{a}\\ \frac{dL}{dt}=\left[\sum_{i}f(M_{i};k,\gamma )n_{i}\lambda_{i}\right]\\ -\left[\mu_{L}+\sum_{i}\beta_{i}n_{i}\right]L;\text{ for }i=p,c,a \end{array}\}$$

From the model, the quantity (*L*) denotes the per capita infectiousness of the shared reservoir, the parameter μ denotes the mortality rate of the mature worms in the host, and β_*i*_ for *i* = *p, c, a* denotes the strength of infectious contact with the reservoir for each age group. The quantity λ_*i*_ for *i* = *p, c, a* describes the relative per capita contributions of infectious materials by each age group. The parameter *n*_*i*_ for *i* = *p, c, a* gives the proportion of the population in each age group. The parameter μ_*L*_ gives the rate of decay of infectious materials in the environment.

The function *f*(*M*_*i*_; *k*, γ) gives the mean egg production rate from a host population with mean worm burden (*M*) and assumed to follow a negative binomial distribution with aggregation parameter (*k*). As described by Anderson and May (29), the function has the form,

$$\begin{array}{l} f\left(M;k,\gamma \right)=\frac{M}{{\left[1+\frac{M}{k}\left(1-e^{-\gamma }\right)\right]}^{k+1}} \end{array}$$

The parameter, exp(−γ), describes the impact of the density-dependence egg production on host's worm burden (24).Further, model 1, as it is, ignores the effect of sexual reproduction and assumes that all eggs generated by female worms in each host group are fertile (non-sexual reproduction). However, in reality, the production of fertile eggs by female worms requires the presence of at least one mature male worm. Hence, to include the effect of sexual reproduction in model 1, we multiply the egg production function, *f*(*M*; *k*, γ), by the mating probability factor ψ, as was given by Truscott et al. (28),

$$\begin{array}{l} \psi =1-{\left[\frac{1+\frac{M}{k}\left(1-e^{-\gamma }\right)}{1+\frac{M}{k}\left(2-e^{-\gamma }\right)}\right]}^{k+1} \end{array}$$

#### 2.3.2. Model With MDA Intervention Only

The second model is when MDA intervention was assumed and it culminated into a four-dimensional system of ODE as follows. We modified the above model 1 by introducing treatment effect denoted as, *c*_*i*_ = −*ln*(1 − *g*_*i*_*h*)/τ, to examine the impact of MDA on mean worm burden and egg output for each age group. Here, *g*_*i*_ denoted the proportion of individuals treated in each age group per treatment round, *h* the drug efficacy, and τ the interval between treatment rounds. Actually, the term *g*_*i*_*h* represented the effective treatment coverage for each age group. This treatment impact equation has been previously used in other STH models (28, 31). It is important to note that we assumed a continuous model for the impact of a long sequence of regular treatment cycles. Hence, the impact of this assumption is analogous to an additional death rate for the parasite population.

(2) $$\begin{array}{l} \frac{dM_{p}}{dt}=\beta_{p}L-(\mu +c_{p})M_{p}\\ \frac{dM_{c}}{dt}=\beta_{c}L-(\mu +c_{c})M_{c}\\ \frac{dM_{a}}{dt}=\beta_{a}L-(\mu +c_{a})M_{a}\\ \frac{dL}{dt}=\left[\sum_{i}f(M_{i};k,\gamma )n_{i}\lambda_{i}\right]\\ -\left[\mu_{L}+\sum_{i}\beta_{i}n_{i}\right]L;\text{ for }i=p,c,a \end{array}\}$$

#### 2.3.3. Model With MDA and WASH Interventions

The third model is when both MDA and WASH interventions were assumed and it culminated into a four-dimensional system of ODE as follows. Again, we modified the above model 1 by introducing both the treatment effect, *c*_*i*_, and WASH effect, ϕ, for 0 ≤ ϕ ≤ 1. Here, we assumed a cumulative effect of WASH defined as the proportion of individuals in each group of the population who have access to and use of improved water sources and sanitation facilities.

(3) $$\begin{array}{l} \frac{dM_{p}}{dt}=\beta_{p}(1-\phi )L-(\mu +c_{p})M_{p}\\ \frac{dM_{c}}{dt}=\beta_{c}(1-\phi )L-(\mu +c_{c})M_{c}\\ \frac{dM_{a}}{dt}=\beta_{a}(1-\phi )L-(\mu +c_{a})M_{a}\\ \frac{dL}{dt}=\left[(1-\phi )\sum_{i}f(M_{i};k,\gamma )n_{i}\lambda_{i}\right]\\ -\left[\mu_{L}+(1-\phi )\sum_{i}\beta_{i}n_{i}\right]L;\text{ for }i=p,c,a \end{array}\}$$

### 2.4. Model Outcome Measures

In this analysis, we were interested in predicting the mean worm burden levels in each of the host's age group over a period of time and the transmission interruption period (elimination). The behavior of the outcomes were assessed based on the different mix and plans of the projected implementation of key interventions (MDA and WASH).

### 2.5. Equilibria and Basic Reproduction Number

Here, we show the calculations of our model equilibrium values defined as the solution of a dynamical system where the state variables does not change with time (37), and the basic reproduction number *R*_0_ defined as the average number of new parasite offsprings caused by one typical parasite, from one generation to the next (38). Generally, if *R*_0_ > 1 then there will be epidemic and if *R*_0_ < 1 the infection will die off. We used the next-generation matrix (NGM) approach to determine *R*_0_ for each of the three models (1, 2, and 3). The matrix related the numbers of new adult worms in consecutive generations. With this approach, then *R*_0_ was defined as the largest eigenvalue of NGM or spectral radius.

#### 2.5.1. Calculation of *R*_0_ and Equilibrium Values for Model 1

**Calculating *R*_0_ for model 1:** From model (1) we obtain the following and with the assumption that *f*(*M*_*i*_; *k*, γ) = *M*_*i*_;

(4) $$\begin{array}{l} \frac{d}{dt}\left[\begin{array}{c} M_{p}\\ M_{c}\\ M_{a}\\ L \end{array}\right]=\mu \left[\begin{array}{cccc} -1 & 0 & 0 & \frac{\beta_{p}}{\mu }\\ 0 & -1 & 0 & \frac{\beta_{c}}{\mu }\\ 0 & 0 & -1 & \frac{\beta_{a}}{\mu }\\ \frac{A}{\mu } & \frac{B}{\mu } & \frac{C}{\mu } & -\frac{\left(D+\mu_{L}\right)}{\mu } \end{array}\right]\;\left[\begin{array}{c} M_{p}\\ M_{c}\\ M_{a}\\ L \end{array}\right] \end{array}$$

We then extract the NGM as follows;

(5) $$\begin{array}{l} \left[\begin{array}{cccc} -1 & 0 & 0 & E\\ 0 & -1 & 0 & F\\ 0 & 0 & -1 & G\\ I & J & K & -H \end{array}\right] \end{array}$$

Where *A* = *n*_*p*_λ_*p*_, *B* = *n*_*c*_λ_*c*_, *C* = *n*_*a*_λ_*a*_, *D* = (*n*_*p*_λ_*p*_+*n*_*c*_λ_*c*_+*n*_*a*_λ_*a*_), $E=\frac{\beta_{p}}{\mu }$, $F=\frac{\beta_{c}}{\mu }$, $G=\frac{\beta_{a}}{\mu }$, $H=\frac{\left(D+\mu_{L}\right)}{\mu }$, $I=\frac{A}{\mu }$, $J=\frac{B}{\mu }$, and $K=\frac{C}{\mu }$ Solving the eigenvalues *u*_*i*_ of NGM (5) using *det*(*NGM* − *uI*) = 0 gives the following eigenvalues;

$$\begin{array}{l} u_{1}=1,\\ u_{2}=-1,\\ u_{3}=-1,\text{and}\\ u_{4}=\left(H-KG-JF-IE\right) \end{array}$$

Therefore, the *R*_0_ is given as the spectral radius of NGM,

(6) $$\begin{array}{l} \left(H-KG-JF-IE\right) \end{array}$$

and simplified as follows;

$$\begin{array}{l} \left[\frac{(\beta_{p}n_{p}+\beta_{c}n_{c}+\beta_{a}n_{a})+\mu_{L}}{\mu }\right]-\left[\left(\frac{n_{a}\lambda_{a}}{\mu }\right)\left(\frac{\beta_{a}}{\mu }\right)\right]\\ -\left[\left(\frac{n_{c}\lambda_{c}}{\mu }\right)\left(\frac{\beta_{c}}{\mu }\right)\right]-\left[\left(\frac{n_{p}\lambda_{p}}{\mu }\right)\left(\frac{\beta_{p}}{\mu }\right)\right]\\ \frac{1}{\mu }[(\beta_{p}n_{p}+\beta_{c}n_{c}+\beta_{a}n_{a})+\mu_{L}-\frac{1}{\mu }(\beta_{p}n_{p}\lambda_{p}+\beta_{c}n_{c}\lambda_{c}\\ \frac{1}{\mu }+\beta_{a}n_{a}\lambda_{a})]\\ \frac{\beta_{p}n_{p}\lambda_{p}+\beta_{c}n_{c}\lambda_{c}+\beta_{a}n_{a}\lambda_{a}}{\mu \left[\mu_{L}+(\beta_{p}n_{p}+\beta_{c}n_{c}+\beta_{a}n_{a})\right]} \end{array}$$

Therefore, the overall *R*_0_ for model (1) is given by;

(7) $$\begin{array}{l} R_{0}=\frac{\beta_{p}n_{p}\lambda_{p}+\beta_{c}n_{c}\lambda_{c}+\beta_{a}n_{a}\lambda_{a}}{\mu \left[\mu_{L}+\left(\beta_{p}n_{p}+\beta_{c}n_{c}+\beta_{a}n_{a}\right)\right]} \end{array}$$

Hence, the *R*_0*i*_ for each age group is a given as;

(8) $$\begin{array}{l} R_{0i}=\frac{\sum_{i}\beta_{i}n_{i}\lambda_{i}}{\mu \left[\mu_{L}+\sum_{i}\beta_{i}n_{i}\right]};\text{ for }i=p,c,a \end{array}$$

**Calculating equilibrium values for model 1:** The relevant equilibrium values were obtained by solving the generalized equations in (1) when the left hand side (LHS) was equated to zero.

In this case, the equilibrium mean infectious materials in the environment (L^*^) is given as

$$\begin{array}{l} \left[f\left(M_{p};k,\gamma \right)n_{p}\lambda_{p}+f\left(M_{c};k,\gamma \right)n_{c}\lambda_{c}+f\left(M_{a};k,\gamma \right)n_{a}\lambda_{a}\right]\\ -\left[\mu_{L}+\left(\beta_{p}n_{p}+\beta_{c}n_{c}+\beta_{a}n_{a}\right)\right]L=0\\ \left[\underset{i}{\sum }f\left(M_{i};k,\gamma \right)n_{i}\lambda_{i}\right]-\left[\mu_{L}+\underset{i}{\sum }\beta_{i}n_{i}\right]L=0 \end{array}$$

Therefore;

(9) $$\begin{array}{l} L^{*}=\frac{\sum_{i}f\left(M_{i};k,\gamma \right)n_{i}\lambda_{i}}{\left[\mu_{L}+\sum_{i}\beta_{i}n_{i}\right]};\text{ for }i=p,c,a \end{array}$$

Subsequently, the equilibrium mean worm burden ($M_{i}^{*}$) was derived by substituting Equation (8) and (9) into (1),

$$\begin{array}{l} \frac{dM_{i}}{dt}=\beta_{i}L^{*}-\mu M_{i}=\beta_{i}\left[\frac{\sum_{i}f\left(M_{i};k,\gamma \right)n_{i}\lambda_{i}}{\mu_{L}+\sum_{i}\beta_{i}n_{i}}\right]-\mu M_{i}\\ \text{But;}\\ \left[\mu_{L}+\underset{i}{\sum }\beta_{i}n_{i}\right]=\frac{\sum_{i}\beta_{i}n_{i}\lambda_{i}}{\mu R_{0i}}\\ \text{Therefore;}\\ \frac{dM_{i}}{dt}=\beta_{i}\left[\frac{\mu R_{0i}\sum_{i}f\left(M_{i};k,\gamma \right)n_{i}\lambda_{i}}{\sum_{i}\beta_{i}n_{i}\lambda_{i}}\right]-\mu M_{i}\\ =\frac{\mu \beta_{i}\sum_{i}R_{0i}f\left(M_{i};k,\gamma \right)n_{i}}{\sum_{i}\beta_{i}n_{i}}-\mu M_{i} \end{array}$$

Hence,

(10) $$\begin{array}{l} \frac{dM_{i}}{dt}=\frac{\mu \beta_{i}\sum_{i}R_{0i}f\left(M_{i};k,\gamma \right)n_{i}}{\sum_{i}\beta_{i}n_{i}}-\mu M_{i} \end{array}$$

We therefore get $M_{i}^{*}$ by setting (10) to zero,

$$\begin{array}{l} \frac{\mu \beta_{i}\sum_{i}R_{0i}f\left(M_{i};k,\gamma \right)n_{i}}{\sum_{i}\beta_{i}n_{i}}-\mu M_{i}=0\\ \mu M_{i}=\frac{\mu \beta_{i}\sum_{i}R_{0i}f\left(M_{i};k,\gamma \right)n_{i}}{\sum_{i}\beta_{i}n_{i}} \end{array}$$

(11) $$\begin{array}{l} M_{i}^{*}=\frac{\beta_{i}\sum_{i}R_{0i}f\left(M_{i};k,\gamma \right)n_{i}}{\sum_{i}\beta_{i}n_{i}};\text{ for }i=p,c,a \end{array}$$

#### 2.5.2. Calculation of *R*_0_ and Equilibrium Values for Model 2

**Calculating *R*_0_ for model 2:** From model (2) we obtain the following and again with the assumption that *f*(*M*_*i*_; *k*, γ) = *M*_*i*_;

(12) $$\begin{array}{l} \frac{d}{dt}\left[\begin{array}{c} M_{p}\\ M_{c}\\ M_{a}\\ L \end{array}\right]=\left(\mu +c_{i}\right)\left[\begin{array}{cccc} -1 & 0 & 0 & \frac{\beta_{p}}{\left(\mu +c_{p}\right)}\\ 0 & -1 & 0 & \frac{\beta_{c}}{\left(\mu +c_{c}\right)}\\ 0 & 0 & -1 & \frac{\beta_{a}}{\left(\mu +c_{a}\right)}\\ \frac{A}{\left(\mu +c_{p}\right)} & \frac{B}{\left(\mu +c_{c}\right)} & \frac{C}{\left(\mu +c_{a}\right)} & -\frac{\left(D+\mu_{L}\right)}{\left(\mu +\sum_{i}c_{i}\right)} \end{array}\right]\;\left[\begin{array}{c} M_{p}\\ M_{c}\\ M_{a}\\ L \end{array}\right] \end{array}$$

We then extract the NGM as follows;

(13) $$\begin{array}{l} \left[\begin{array}{cccc} -1 & 0 & 0 & E\\ 0 & -1 & 0 & F\\ 0 & 0 & -1 & G\\ I & J & K & -H \end{array}\right] \end{array}$$

Where *A* = *n*_*p*_λ_*p*_, *B* = *n*_*c*_λ_*c*_, *C* = *n*_*a*_λ_*a*_, *D* = (*n*_*p*_λ_*p*_+*n*_*c*_λ_*c*_+*n*_*a*_λ_*a*_), $E=\frac{\beta_{p}}{\left(\mu +c_{p}\right)}$, $F=\frac{\beta_{c}}{\left(\mu +c_{c}\right)}$, $G=\frac{\beta_{a}}{\left(\mu +c_{a}\right)}$, $H=\frac{\left(\mu_{L}+D\right)}{\left(\mu +\underset{i}{\sum }c_{i}\right)}$, $I=\frac{A}{\left(\mu +c_{p}\right)}$, $J=\frac{B}{\left(\mu +c_{c}\right)}$, and $K=\frac{C}{\left(\mu +c_{a}\right)}$

But the matrix (13) above is the same as that given in (5), and its spectral radius was (*H*−*KG*−*JF*−*IE*), we then subsequently derive *R*_0_ as,

$$\begin{array}{l} \frac{1}{\left(\mu +\sum_{i}c_{i}\right)}[(\beta_{p}n_{p}+\beta_{c}n_{c}+\beta_{a}n_{a})+\mu_{L}\\ -\frac{1}{\left(\mu +\sum_{i}c_{i}\right)}(\beta_{p}n_{p}\lambda_{p}+\beta_{c}n_{c}\lambda_{c}+\beta_{a}n_{a}\lambda_{a})]\\ \frac{\beta_{p}n_{p}\lambda_{p}+\beta_{c}n_{c}\lambda_{c}+\beta_{a}n_{a}\lambda_{a}}{(\mu +\sum_{i}c_{i})\left[\mu_{L}+(\beta_{p}n_{p}+\beta_{c}n_{c}+\beta_{a}n_{a})\right]} \end{array}$$

Therefore, the overall *R*_0_ for model (2) is given by;

(14) $$\begin{array}{l} R_{0}=\frac{\beta_{p}n_{p}\lambda_{p}+\beta_{c}n_{c}\lambda_{c}+\beta_{a}n_{a}\lambda_{a}}{\left(\mu +\sum_{i}c_{i}\right)\left[\mu_{L}+\left(\beta_{p}n_{p}+\beta_{c}n_{c}+\beta_{a}n_{a}\right)\right]} \end{array}$$

Hence, the *R*_0*i*_ for each age group is a given as;

(15) $$\begin{array}{l} R_{0i}=\frac{\sum_{i}\beta_{i}n_{i}\lambda_{i}}{\left(\mu +c_{i}\right)\left[\mu_{L}+\sum_{i}\beta_{i}n_{i}\right]};\text{for}i=p,c,a \end{array}$$

**Calculating equilibrium values for model 2:** The relevant equilibrium values for model 2 were obtained by solving the generalized equations in (2) when the LHS was equated to zero.

Using the same procedure described for model 1, it is easy to see that the equalibrium function for (L^*^) is the same as that of Equation (9).

We then obtain equalibrium mean worm burden ($M_{i}^{*}$) by substituting (9) and (15) into (2),

$$\begin{array}{l} \frac{dM_{i}}{dt}=\beta_{i}L^{*}-\left(\mu +c_{i}\right)M_{i}=\beta_{i}\left[\frac{\sum_{i}f\left(M_{i};k,\gamma \right)n_{i}\lambda_{i}}{\mu_{L}+\sum_{i}\beta_{i}n_{i}}\right]\\ -\left(\mu +c_{i}\right)M_{i}\\ \text{But;}\\ \left[\mu_{L}+\underset{i}{\sum }\beta_{i}n_{i}\right]=\frac{\sum_{i}\beta_{i}n_{i}\lambda_{i}}{\left(\mu +c_{i}\right)R_{0i}}\\ \frac{dM_{i}}{dt}=\beta_{i}\left[\frac{\left(\mu +c_{i}\right)R_{0i}\sum_{i}f\left(M_{i};k,\gamma \right)n_{i}\lambda_{i}}{\sum_{i}\beta_{i}n_{i}\lambda_{i}}\right]-\left(\mu +c_{i}\right)M_{i}\\ =\frac{\left(\mu +c_{i}\right)\beta_{i}\sum_{i}R_{0i}f\left(M_{i};k,\gamma \right)n_{i}}{\sum_{i}\beta_{i}n_{i}}-\left(\mu +c_{i}\right)M_{i} \end{array}$$

Hence,

(16) $$\begin{array}{l} \frac{dM_{i}}{dt}=\frac{\left(\mu +c_{i}\right)\beta_{i}\sum_{i}R_{0i}f\left(M_{i};k,\gamma \right)n_{i}}{\sum_{i}\beta_{i}n_{i}}-\left(\mu +c_{i}\right)M_{i} \end{array}$$

and ($M_{i}^{*}$) is given as,

(17) $$\begin{array}{l} M_{i}^{*}=\frac{\beta_{i}\sum_{i}R_{0i}f\left(M_{i};k,\gamma \right)n_{i}}{\sum_{i}\beta_{i}n_{i}};\text{ for }i=p,c,a \end{array}$$

which is the same as that given in (11).

#### 2.5.3. Calculation of *R*_0_ and Equilibrium Values for Model 3

**Calculating *R*_0_ for model 3:** From model (3) we obtain the following and again with the assumption that *f*(*M*_*i*_; *k*, γ) = *M*_*i*_;

(18) $$\begin{array}{l} \frac{d}{dt}\left[\begin{array}{c} M_{p}\\ M_{c}\\ M_{a}\\ L \end{array}\right]=\left(\mu +c_{i}\right)\left[\begin{array}{cccc} -1 & 0 & 0 & \frac{\beta_{p}\left(1-\phi \right)}{\left(\mu +c_{p}\right)}\\ 0 & -1 & 0 & \frac{\beta_{c}\left(1-\phi \right)}{\left(\mu +c_{c}\right)}\\ 0 & 0 & -1 & \frac{\beta_{a}\left(1-\phi \right)}{\left(\mu +c_{a}\right)}\\ \frac{A}{\left(\mu +c_{p}\right)} & \frac{B}{\left(\mu +c_{c}\right)} & \frac{C}{\left(\mu +c_{a}\right)} & -\frac{\left(D+\mu_{L}\right)}{\left(\mu +\sum_{i}c_{i}\right)} \end{array}\right]\;\left[\begin{array}{c} M_{p}\\ M_{c}\\ M_{a}\\ L \end{array}\right] \end{array}$$

We then extract the NGM as follows;

(19) $$\begin{array}{l} \left[\begin{array}{cccc} -1 & 0 & 0 & E\\ 0 & -1 & 0 & F\\ 0 & 0 & -1 & G\\ I & J & K & -H \end{array}\right] \end{array}$$

Where *A* = *n*_*p*_λ_*p*_(1 − ϕ), *B* = *n*_*c*_λ_*c*_(1 − ϕ), *C* = *n*_*a*_λ_*a*_(1 − ϕ), *D* = (*n*_*p*_λ_*p*_(1 − ϕ) + *n*_*c*_λ_*c*_(1 − ϕ) + *n*_*a*_λ_*a*_(1 − ϕ)), $E=\frac{\beta_{p}\left(1-\phi \right)}{\left(\mu +c_{p}\right)}$, $F=\frac{\beta_{c}\left(1-\phi \right)}{\left(\mu +c_{c}\right)}$, $G=\frac{\beta_{a}\left(1-\phi \right)}{\left(\mu +c_{a}\right)}$, $H=\frac{\left(\mu_{L}+D\right)}{\left(\mu +\underset{i}{\sum }c_{i}\right)}$, $I=\frac{A}{\left(\mu +c_{p}\right)}$, $J=\frac{B}{\left(\mu +c_{c}\right)}$, and $K=\frac{C}{\left(\mu +c_{a}\right)}$

But the matrix (19) above is the same as that given in (5), and its spectral radius was (*H* − *KG* − *JF* − *IE*), we then subsequently derive *R*_0_ as,

$$\begin{array}{l} \frac{1}{\left(\mu +\sum_{i}c_{i}\right)}[(1-\phi )(\beta_{p}n_{p}+\beta_{c}n_{c}+\beta_{a}n_{a})+\mu_{L}\\ -\frac{{\left(1-\phi \right)}^{2}}{\left(\mu +\sum_{i}c_{i}\right)}(\beta_{p}n_{p}\lambda_{p}+\beta_{c}n_{c}\lambda_{c}+\beta_{a}n_{a}\lambda_{a})]\\ \frac{{\left(1-\phi \right)}^{2}(\beta_{p}n_{p}\lambda_{p}+\beta_{c}n_{c}\lambda_{c}+\beta_{a}n_{a}\lambda_{a})}{(\mu +\sum_{i}c_{i})\left[\mu_{L}+(1-\phi )(\beta_{p}n_{p}+\beta_{c}n_{c}+\beta_{a}n_{a})\right]} \end{array}$$

Therefore, the overall *R*_0_ for model (3) is given by;

(20) $$\begin{array}{l} R_{0}=\frac{{\left(1-\phi \right)}^{2}\left(\beta_{p}n_{p}\lambda_{p}+\beta_{c}n_{c}\lambda_{c}+\beta_{a}n_{a}\lambda_{a}\right)}{\left(\mu +\sum_{i}c_{i}\right)\left[\mu_{L}+\left(1-\phi \right)\left(\beta_{p}n_{p}+\beta_{c}n_{c}+\beta_{a}n_{a}\right)\right]} \end{array}$$

Hence, the *R*_0*i*_ for each age group is a given as;

(21) $$\begin{array}{l} R_{0i}=\frac{{\left(1-\phi \right)}^{2}\sum_{i}\beta_{i}n_{i}\lambda_{i}}{\left(\mu +c_{i}\right)\left[\mu_{L}+\left(1-\phi \right)\sum_{i}\beta_{i}n_{i}\right]};\\ \text{ for }i=p,c,a\text{ and }0\leq \phi \leq 1 \end{array}$$

**Calculating equilibrium values for model 3:** The relevant equilibrium values were obtained by solving the generalized equations in (3) when the LHS was equated to zero.

In this case, and using the same procedure as described for model (1), L^*^ is given as

$$\begin{array}{l} \left[\left(1-\phi \right)\underset{i}{\sum }f\left(M_{i};k,\gamma \right)n_{i}\lambda_{i}\right]-\left[\mu_{L}+\left(1-\phi \right)\underset{i}{\sum }\beta_{i}n_{i}\right]L=0 \end{array}$$

Therefore;

(22) $$\begin{array}{l} L^{*}=\frac{\left(1-\phi \right)\sum_{i}f\left(M_{i};k,\gamma \right)n_{i}\lambda_{i}}{\left[\mu_{L}+\left(1-\phi \right)\sum_{i}\beta_{i}n_{i}\right]};\\ \text{for }i=p,c,a\text{ and }0\leq \phi \leq 1 \end{array}$$

We then obtain equalibrium mean worm burden ($M_{i}^{*}$) by substituting (21) and (22) into (3),

$$\begin{array}{l} \frac{dM_{i}}{dt}=\beta_{i}\left(1-\phi \right)L^{*}-\left(\mu +c_{i}\right)M_{i}\\ =\beta_{i}\left(1-\phi \right)\left[\frac{\left(1-\phi \right)\sum_{i}f\left(M_{i};k,\gamma \right)n_{i}\lambda_{i}}{\left[\mu_{L}+\left(1-\phi \right)\sum_{i}\beta_{i}n_{i}\right]}\right]-\left(\mu +c_{i}\right)M_{i}\\ \text{But;}\\ \left[\mu_{L}+\left(1-\phi \right)\underset{i}{\sum }\beta_{i}n_{i}\right]=\frac{{\left(1-\phi \right)}^{2}\sum_{i}\beta_{i}n_{i}\lambda_{i}}{\left(\mu +c_{i}\right)R_{0i}}\\ \frac{dM_{i}}{dt}=\beta_{i}\left(1-\phi \right)\left[\frac{\left(\mu +c_{i}\right)R_{0i}\left(1-\phi \right)\sum_{i}f\left(M_{i};k,\gamma \right)n_{i}\lambda_{i}}{{\left(1-\phi \right)}^{2}\sum_{i}\beta_{i}n_{i}\lambda_{i}}\right]\\ -\left(\mu +c_{i}\right)M_{i}\\ =\frac{\left(\mu +c_{i}\right)\beta_{i}{\left(1-\phi \right)}^{2}\sum_{i}R_{0i}f\left(M_{i};k,\gamma \right)n_{i}}{{\left(1-\phi \right)}^{2}\sum_{i}\beta_{i}n_{i}}-\left(\mu +c_{i}\right)M_{i} \end{array}$$

Hence,

(23) $$\begin{array}{l} \frac{dM_{i}}{dt}=\frac{\left(\mu +c_{i}\right)\beta_{i}{\left(1-\phi \right)}^{2}\sum_{i}R_{0i}f\left(M_{i};k,\gamma \right)n_{i}}{{\left(1-\phi \right)}^{2}\sum_{i}\beta_{i}n_{i}}-\left(\mu +c_{i}\right)M_{i} \end{array}$$

and ($M_{i}^{*}$) is given as,

(24) $$\begin{array}{l} M_{i}^{*}=\frac{\beta_{i}\sum_{i}R_{0i}f\left(M_{i};k,\gamma \right)n_{i}}{\sum_{i}\beta_{i}n_{i}};\text{ for }i=p,c,a \end{array}$$

which is the same as that given in (11).

From the calculations of the mean worm burden for all the models as seen in Equations (11), (17), and (24), we see that the models attained same equilibrium points for the mean worm burden regardless of the incorporated interventions. This imply that if the interventions are not implemented consistently and for long enough period or stopped early, then the infections will rebound (re-infection) and the mean worm burden in the hosts will raise to the initial levels. A concept that has been supported in previous epidemiological and modeling studies (11, 14). However, the maximum attainable *R*_0_ values were observed to vary based on the model considered.

### 2.6. Model Parameter Estimates

The default parameter values used in this analysis are given in Table 1. They represented scenario for all the three STH species; *A. lumbricoides*, hookworm, and *T. trichiura*. Some of the parameters were estimated using field data collected in Kenya, a community where children population, particularly SAC, previously had twice the exposure to infectious materials in the environment compared to adult population. While the remaining parameters were estimated from past modeling and simulation studies. Currently, treatment is offered to SAC through annual MDA program with a net drug (albendazole) efficacy of 80% but this varies by the specific STH species. The output of this model was compared with the five-year Kenyan deworming program results (see subsection 3.1) for all the three STH species.

**Table 1:** Model parameters with default values for each of the three STH species used in all calculations (unless otherwise stated).

**Parameters**	***A. lumbricoides***	**Hookworm**	***T. trichiura***	**Source**
Infection transmission rate among PSAC, β_*p*_	0.91	1.8	0.31	Estimated from Kenyan deworming data
Infection transmission rate among SAC, β_*c*_	0.98	2.2	0.28	(11)
Infection transmission rate among adults, β_*a*_	0.77	2.5	0.25	(26)
Proportion of PSAC in the population, *n*_*p*_	0.05	0.05	0.05	(39)
Proportion of SAC in the population, *n*_*c*_	0.25	0.25	0.25	(39)
Proportion of adults in the population, *n*_*a*_	0.70	0.70	0.70	(39)
Relative contributions of PSAC to the environment, λ_*p*_	2.5	2.0	1.92	Estimated from Kenyan deworming data
Relative contributions of SAC to the environment, λ_*c*_	4.0	3.0	1.92	Estimated from Kenyan deworming data
Relative contributions of adults to the environment, λ_*a*_	3.5	4.0	1.82	Estimated from Kenyan deworming data
Average life span of the mature worm in the host, $\frac{1}{\mu }$	1 year	2 years	2 years	(31)
Average life span of free-living infectious materials, $\frac{1}{\mu_{L}}$	84 days	3 days	10 days	(29)
Strength of density dependence of worm egg production, γ	0.0035	0.01	0.01	(28)
Over-dispersion (aggregation) parameter, *k*	0.57	0.8	0.11	(32)
Treatment effect, *c*_*i*_	Varied, see text	Varied, see text	Varied, see text	see text
WASH effect, ϕ	Varied, see text	Varied, see text	Varied, see text	see text

## 3. Results

### 3.1. Summary of the Kenyan National Deworming Program Results

The Kenyan deworming program conducted yearly cross-sectional surveys before the annual treatment delivery (MDA) to determine the national STH infection levels. The program monitored two key outcomes during their surveys; the mean number of parasite eggs in each participating individual and the infection prevalence (%). From these outcomes, the program was interested in their decline over the years as the annual treatment progresses. Table 2 summarizes the results from the Kenyan program. We note that this program targeted SAC only through SBD program approach. However, adults and PSAC were only treated and sampled during the surveys only when they presented themselves at the nearest participating school.

**Table 2:** Summary of STH surveys in Kenya from 2012 to 2017.

			**Mean number of eggs per gram (epg)**			**Prevalence (%)**		
**Survey year**	**# Schools**	**# Examined**	**Min**	**Max**	**Mean**	**Min**	**Max**	**Mean**
Year 1								
PSAC	44	273	0	18018	1645.9	0	100	28.0
SAC	60	5938	0	8450	1717.5	0.9	61.7	33.5
Adults	51	201	0	14868	983.2	0	100	29.8
Overall	60	6412	0	18018	1455.0	0	100	30.7
Year 2								
PSAC	32	109	0	8736	1245.8	0	100	16.9
SAC	60	6133	0	4829	1090.5	0	51.9	18.7
Adults	47	118	0	24024	914.3	0	100	18.6
Overall	60	6360	0	24024	1066.7	0	100	18.3
Year 3								
PSAC	28	102	0	4776	627.1	0	100	12.6
SAC	60	6029	0	5295	917.2	0	55.2	16.2
Adults	37	121	0	2754	255.8	0	100	15.8
Overall	60	6252	0	5295	656.4	0	100	15.2
Year 4								
PSAC	22	66	0	16002	1814.6	0	100	22.9
SAC	60	6025	0	9009	1210.9	0	58.2	15.8
Adults	27	97	0	1074	87.0	0	100	11.2
Overall	60	6188	0	16002	1054.3	0	100	16.1
Year 5								
PSAC	22	80	0	2523	279.2	0	100	9.7
SAC	60	6074	0	5873	937.2	0	67.9	13.5
Adults	34	84	0	9288	745.5	0	100	11.1
Overall	60	6238	0	9288	756.3	0	100	12.1

*The surveys were conducted through the Kenyan national school-based deworming program which targeted school going children. Pre-school children and adults were essentially surveyed only when they presented themselves to the nearest participating school*.

#### 3.1.1. Mean Number of Eggs

**Figure 2:**
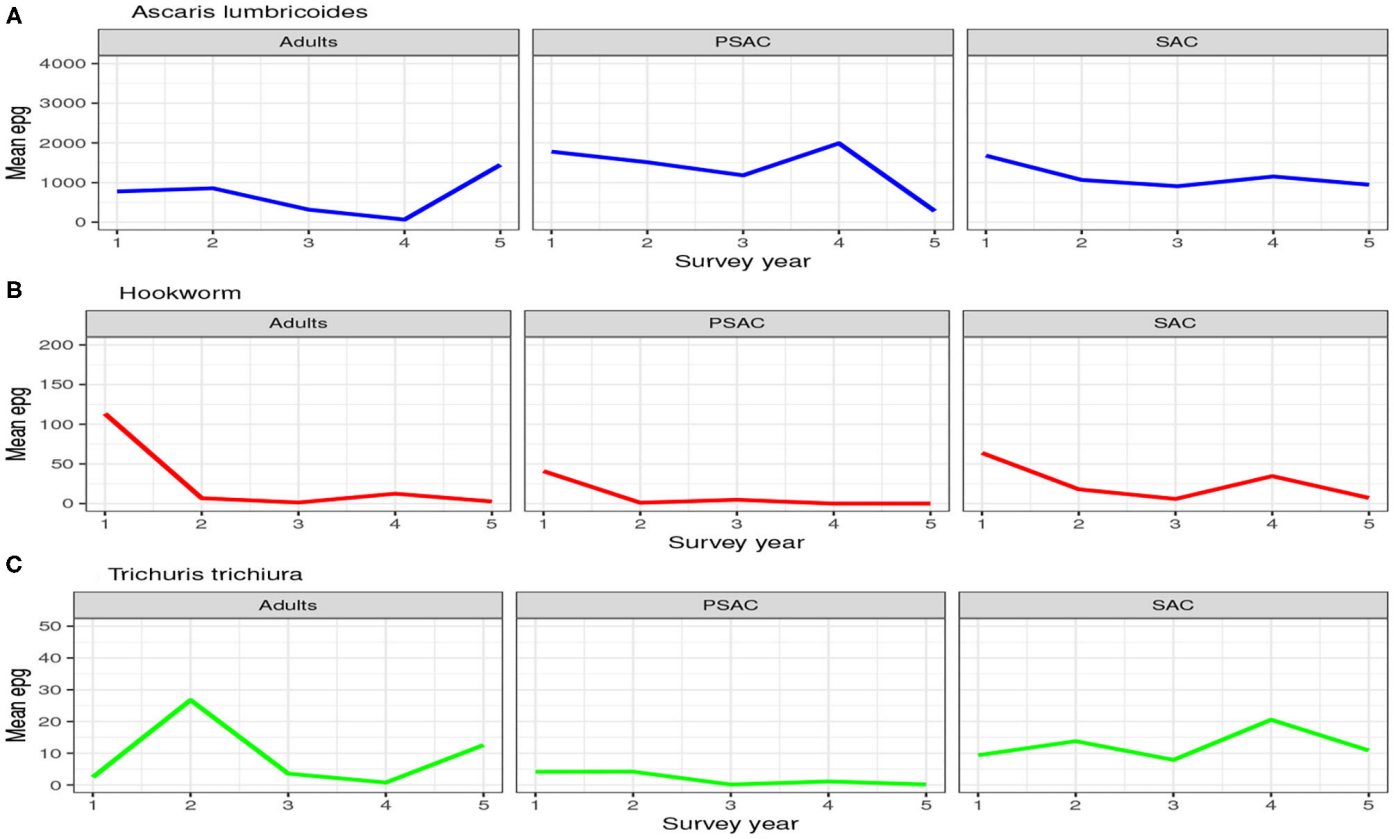
Trend of the hosts mean number of eggs per gram (epg) as observed from the 5-year national deworming program in Kenya. **(A)** *Ascaris lumbricoides*. **(B)** Hookworm. **(C)** *Trichuris trichiura*.

The trend in the mean number of eggs per gram (epg) in each individual surveyed is summarized in Figure 2. From this figure we can conclude that; (1) the mean number of eggs for *A. lumbricoides* only reduced gradually over the years among SAC cohort but not among adults and PSAC, (2) the reduction in the mean number of eggs for hookworm was clearly observed among all the three age groups, and (3) no clear trend of reduction was observed for *T. trichiura* among any of the age groups. Additionally, we noted that there were higher mean number of eggs for *A. lumbricoides* compared to hookworm or *T. trichiura*, which all had lower number of eggs.

#### 3.1.2. Infection Prevalence

**Figure 3:**
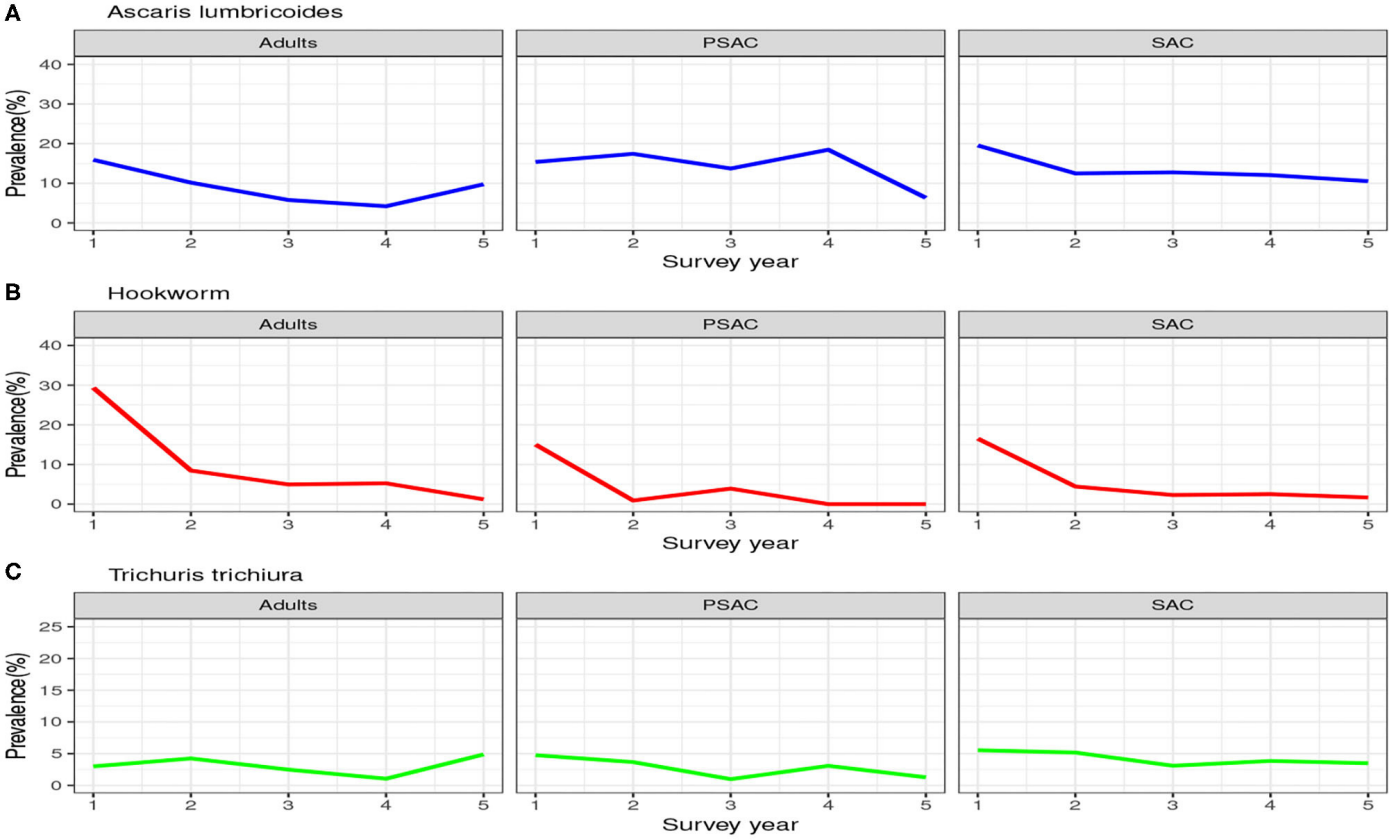
Prevalence trend as observed from the 5-year national deworming program in Kenya. **(A)** *Ascaris lumbricoides*. **(B)** Hookworm. **(C)** *Trichuris trichiura*.

Similarly, the trend in the infection prevalence among surveyed individuals is summarized in Figure 3. From this figure we can conclude that the infection prevalence reduced gradually among SAC but not the other age groups, and the reduction was well-pronounced in hookworm than the other STH species. Additionally, we noted that there were initially higher prevalence for *A. lumbricoides* and hookworm compared to *T. trichiura*. After the 5-year period, hookworm prevalence declined substantially to near-zero compared to the other STH species. This implied greater impact of the annual deworming program on hookworm compared to the other species.

Clearly, the impact of the annual MDA by the Kenyan program was well-pronounced among the SAC compared to PSAC and adults. This is mainly true because the program targeted the SAC age group only. As shown, this program will not likely lead to community-wide infection elimination in the short run especially for *A. lumbricoides* and *T. trichiura*.

### 3.2. Model Results

Here, we give the results of the model simulation based on parameters outlined in Table 1. The model results mimicked closely the STH dynamics in Kenya as observed from the Kenyan deworming program results (subsection 3.1). As opposed to the outcomes surveyed by the Kenyan program, the model simulation was interested in two outcomes i.e., the mean number worms in each age group and the elimination period.

#### 3.2.1. *Ascaris lumbricoides*

**Figure 4:**
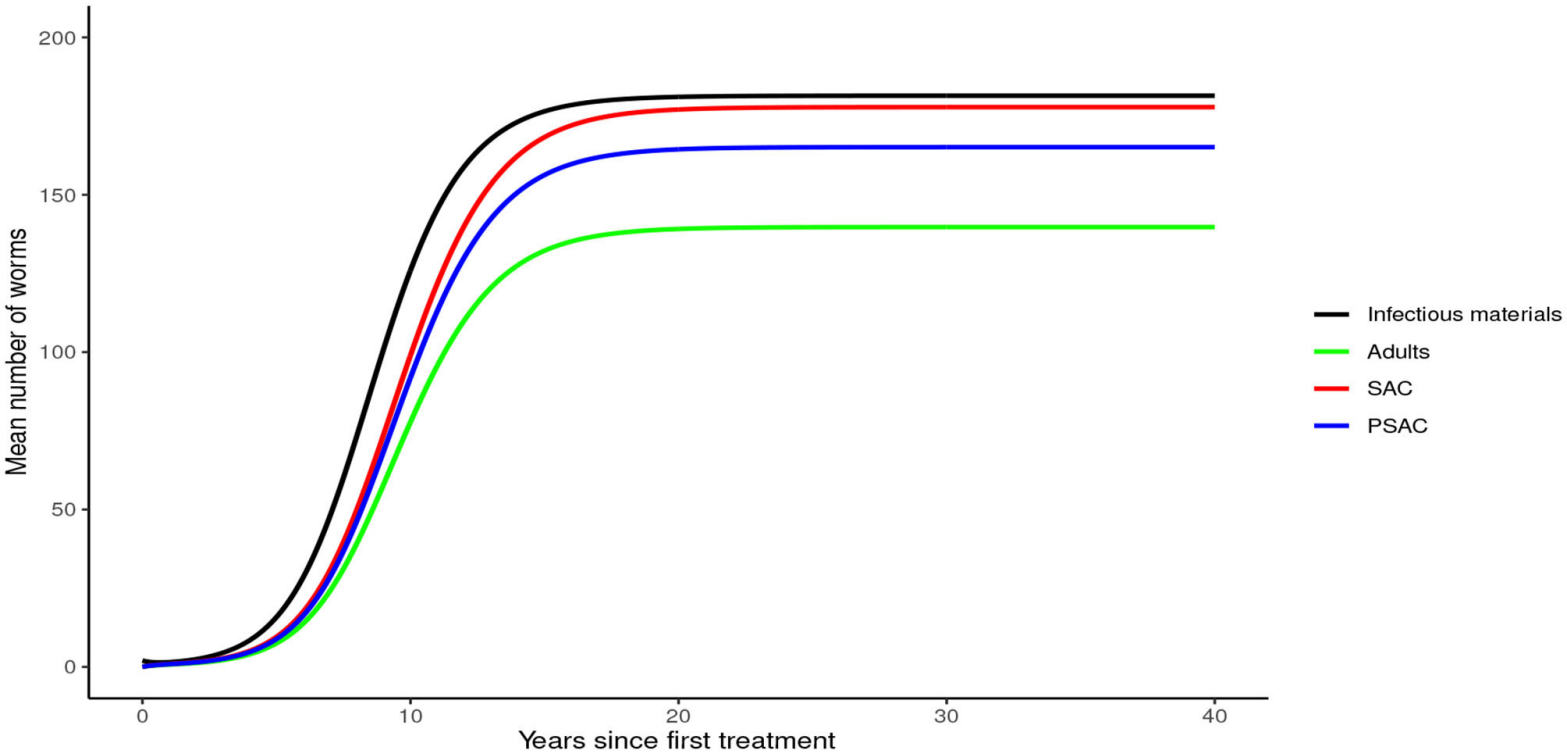
Model 1 solution for *Ascaris lumbricoides*: Here, no intervention was assumed.

Analysis of model (1) for *Ascaris lumbricoides* showed that in the absence of any intervention, the model attains high endemic equilibrium values of varying levels in each compartment, i.e., 170 mean infectious materials in the environment and between 140 to 160 mean worm burden in the human hosts (see Figure 4).

**Figure 5:**
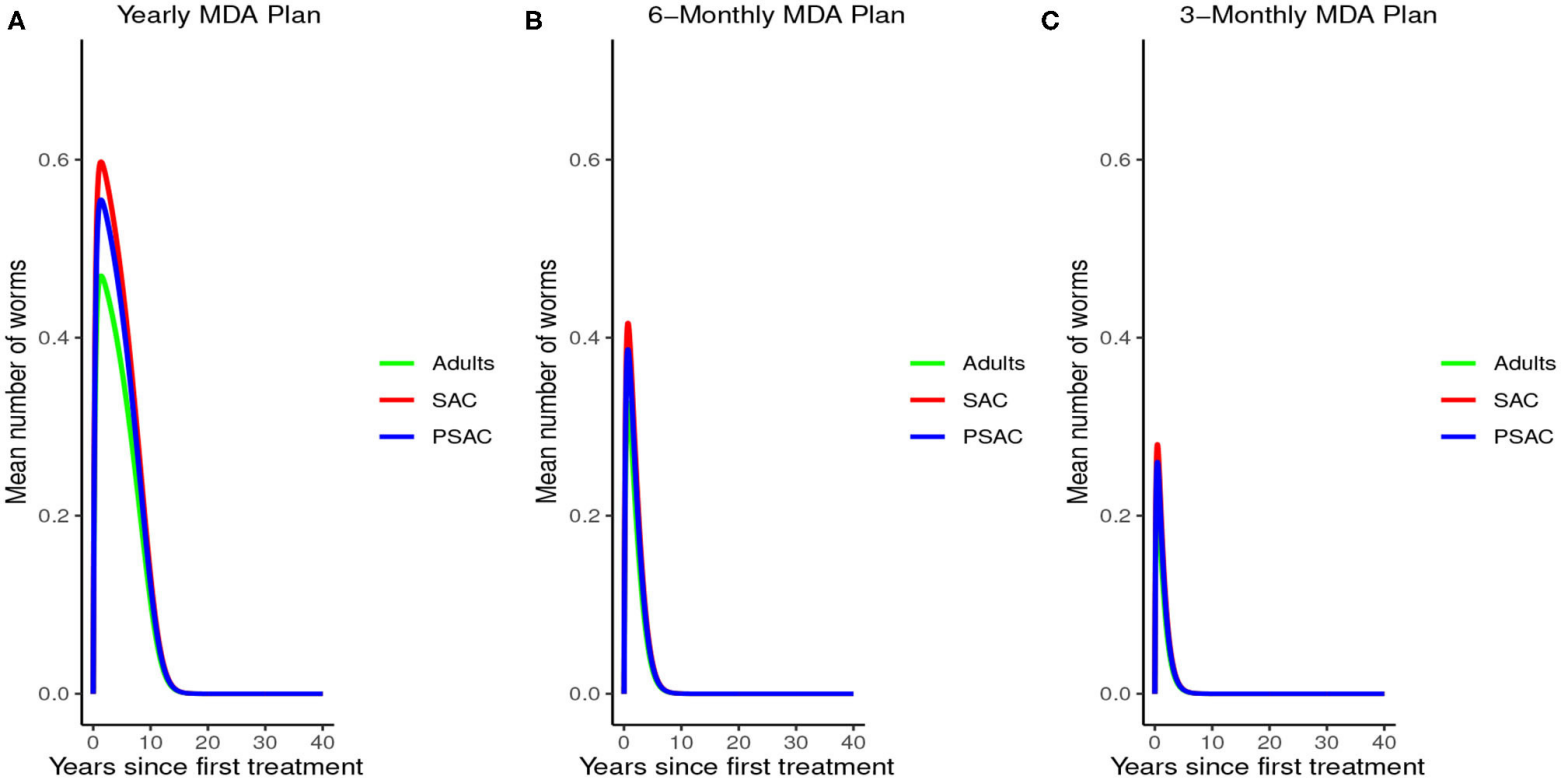
Model 2 solution for *Ascaris lumbricoides*: We assumed various MDA plans as indicated in **(A–C)** i.e., τ = 1.0 for yearly plan, τ = 0.5 for 6-monthly plan, and τ = 0.25 for 3-monthly plan. We assumed treatment coverage of 75% for each host group and drug efficacy (h) of 80%. These assumptions followed the current WHO and NSBD guidelines (13).

Analysis of model (2) for *A. lumbricoides* indicated that elimination of this parasite is possible at various levels of MDA plans. The analysis showed that; with annual MDA plan only (Figure 5A) elimination would be reached after a long period of 15 years. With 6-monthly plan (Figure 5B), elimination point would be reduced to 8 years and endemic worm burden shorten by a third. With 3-monthly plan (Figure 5C), elimination point would be shorten further to 6 years and endemic worm burden reduced by half.

**Figure 6:**
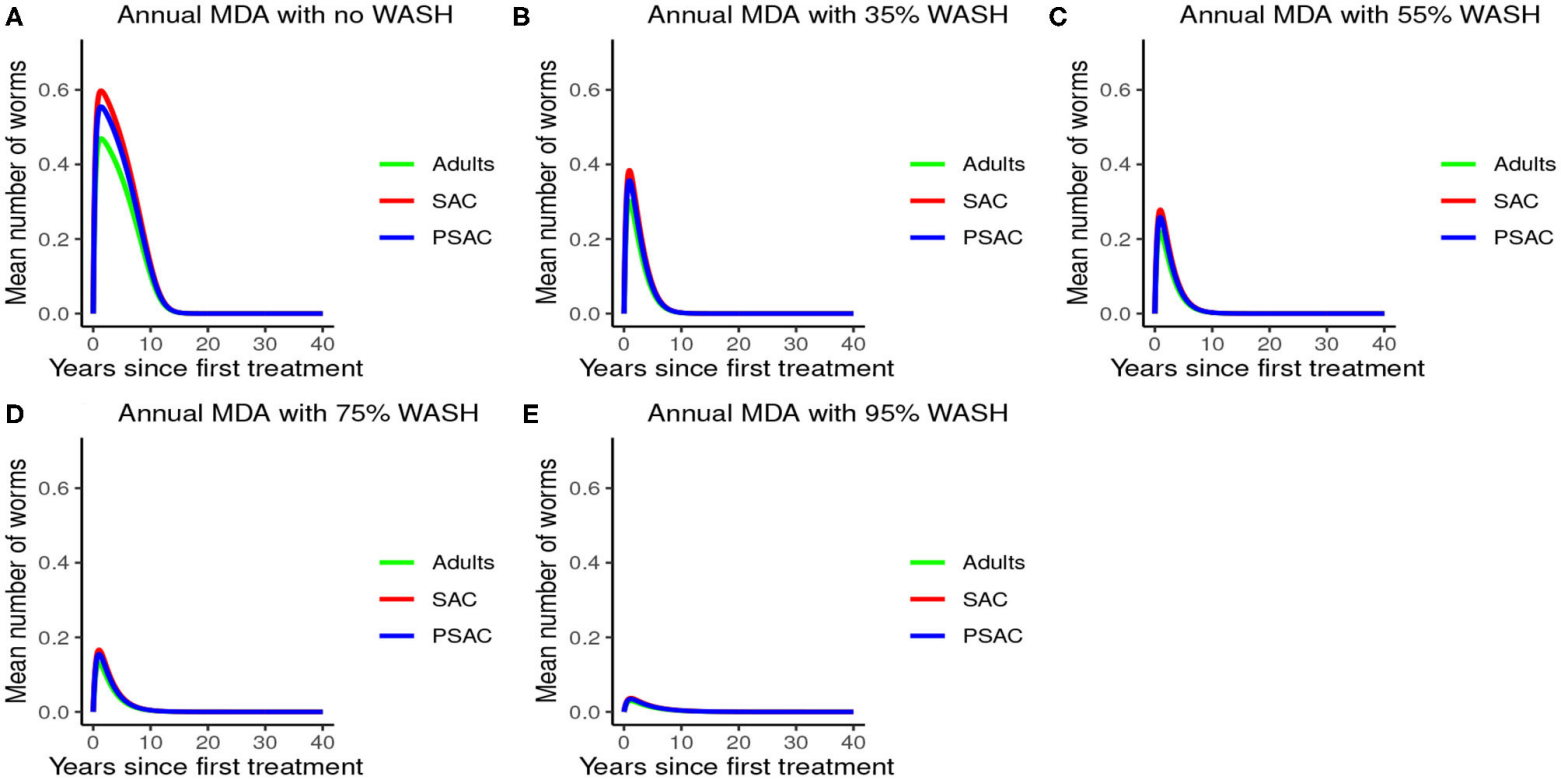
Model 3 solution for *Ascaris lumbricoides*: We assumed annual MDA plan with various WASH levels as indicated in **(A–E)** i.e., ϕ = 0 for no WASH, ϕ = 0.35 for 35% WASH, ϕ = 0.55 for 55% WASH, ϕ = 0.75 for 75% WASH, and ϕ = 0.95 for 95% WASH. Further, we assumed treatment coverage of 75% for each host group and drug efficacy (h) of 80%. These assumptions followed the current WHO and NSBD guidelines (13).

Further, the results indicated that complementing annual MDA with WASH at various coverage levels effectively reduced mean worm burden as WASH coverage increased. Additionally, elimination period was reduced as WASH coverage increased (see Figure 6). Therefore, from the results of model (3), annual MDA complemented with 95% WASH coverage was most effective in shortening the elimination period for *A. lumbricoides*.

#### 3.2.2. Hookworm

**Figure 7:**
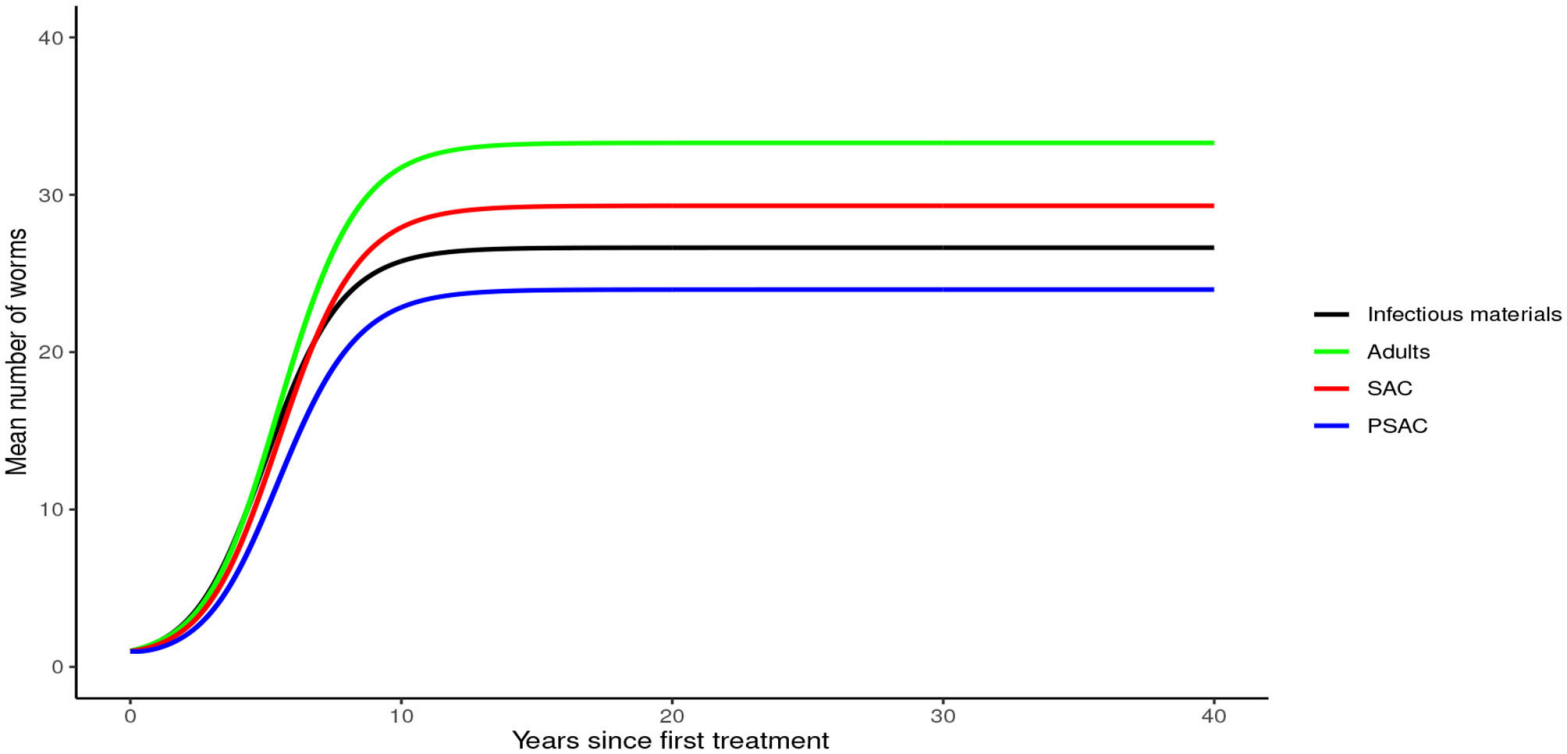
Model 1 solution for hookworm: Here, no intervention was assumed.

In the absence of any interventions, hookworm rose to an endemic equilibrium of between 20 and 35 mean worm burden in the hosts and 25 mean infectious materials in the environment (see Figure 7). Notably, the time taken for hookworm to reach endemic equilibrium level was shorter than for *A. lumbricoides*.

**Figure 8:**
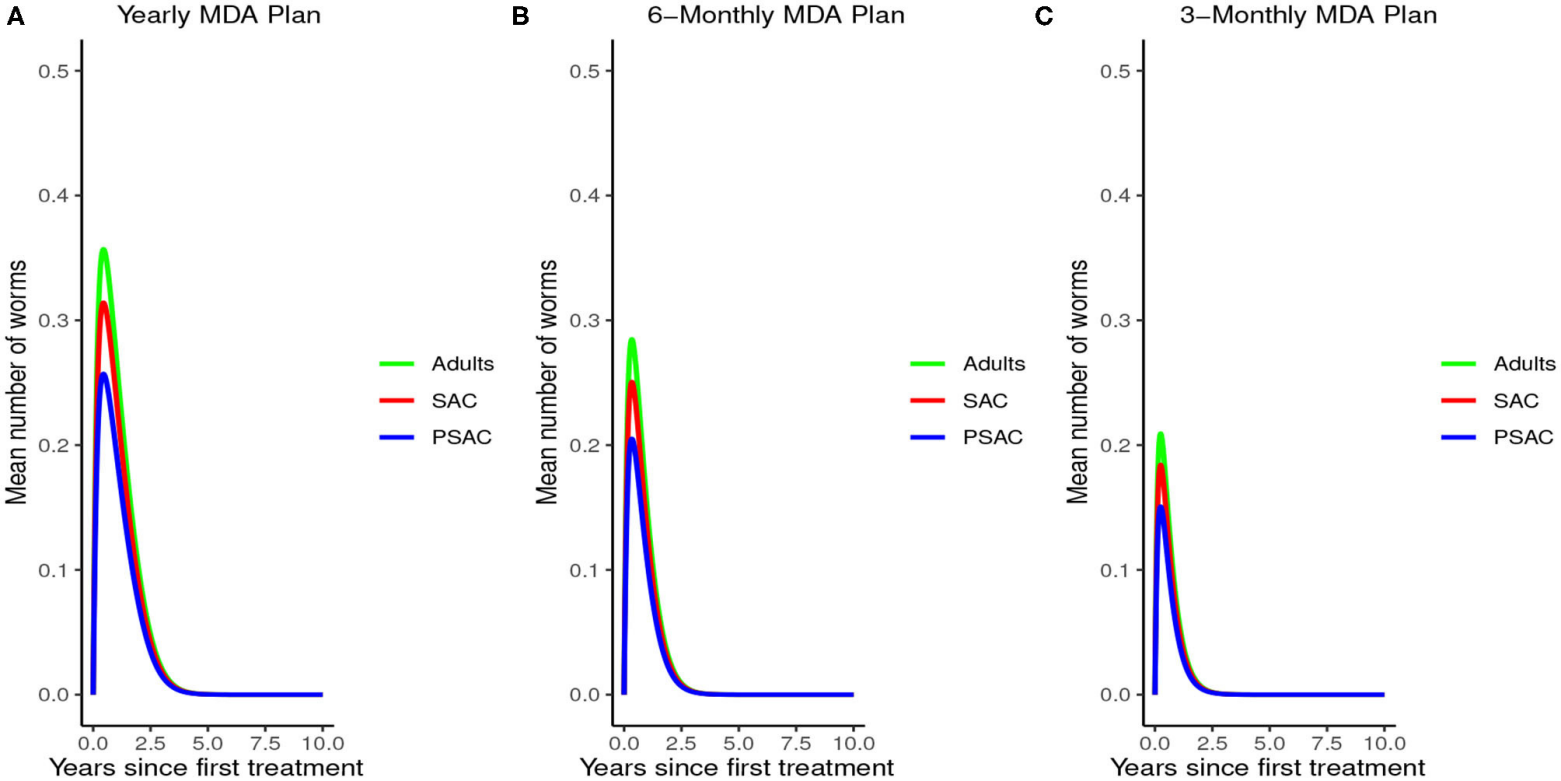
Model 2 solution for hookworm: We assumed various MDA plans as indicated in **(A–C)** i.e., τ = 1.0 for yearly plan, τ = 0.5 for 6-monthly plan, and τ = 0.25 for 3-monthly plan. We assumed treatment coverage of 75% for each host group and drug efficacy (h) of 95%. These assumptions followed the current WHO and NSBD guidelines (13).

Figure 8 demonstrated the impact of MDA on worm burden and elimination period for hookworm. The results indicated that with yearly MDA plan alone (Figure 8A), hookworm infection can be eliminated in less than 5 years. Six-monthly (Figure 8B) and 3-monthly (Figure 8C) plans effectively reduced the endemic worm burden as well as shortened the elimination period. Notably, MDA alone was observed to be effective in eliminating hookworm within a shorter period than in *A. lumbricoides*.

**Figure 9:**
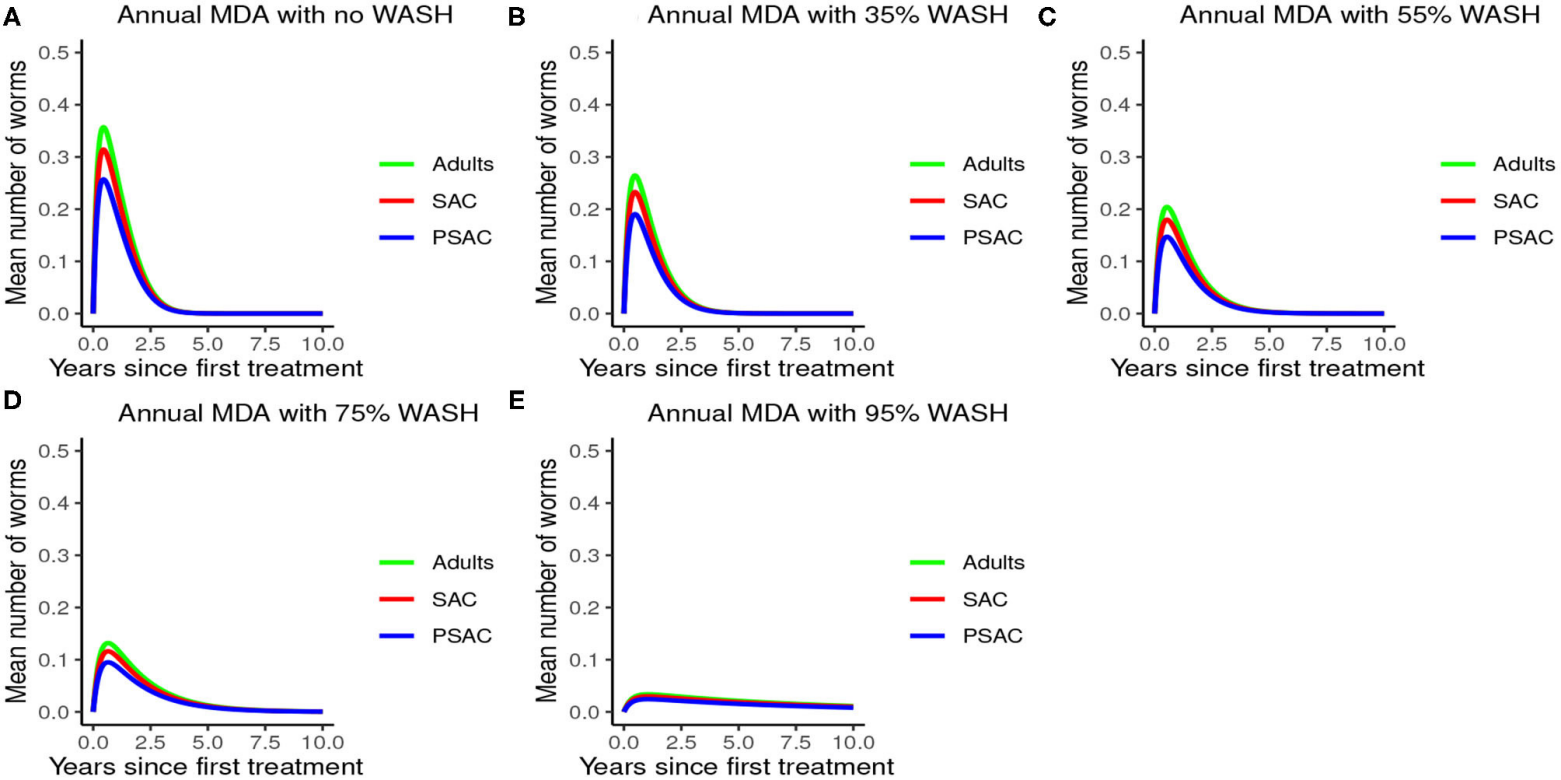
Model 3 solution for hookworm: We assumed annual MDA plan with various WASH levels as indicated in **(A–E)** i.e., ϕ = 0 for no WASH, ϕ = 0.35 for 35% WASH, ϕ = 0.55 for 55% WASH, ϕ = 0.75 for 75% WASH, and ϕ = 0.95 for 95% WASH. Further, we assumed treatment coverage of 75% for each host group and drug efficacy (h) of 95%. These assumptions followed the current WHO and NSBD guidelines (13).

The impact of WASH was clearly observed on reducing the hookworm burden. WASH was most effective when implemented at higher (95%) coverage levels. Complementing annual MDA with WASH (at 95% coverage) was demonstrated to be highly effective in attaining a near-complete elimination of hookworm (see Figure 9).

#### 3.2.3. *Trichuris trichiura*

**Figure 10:**
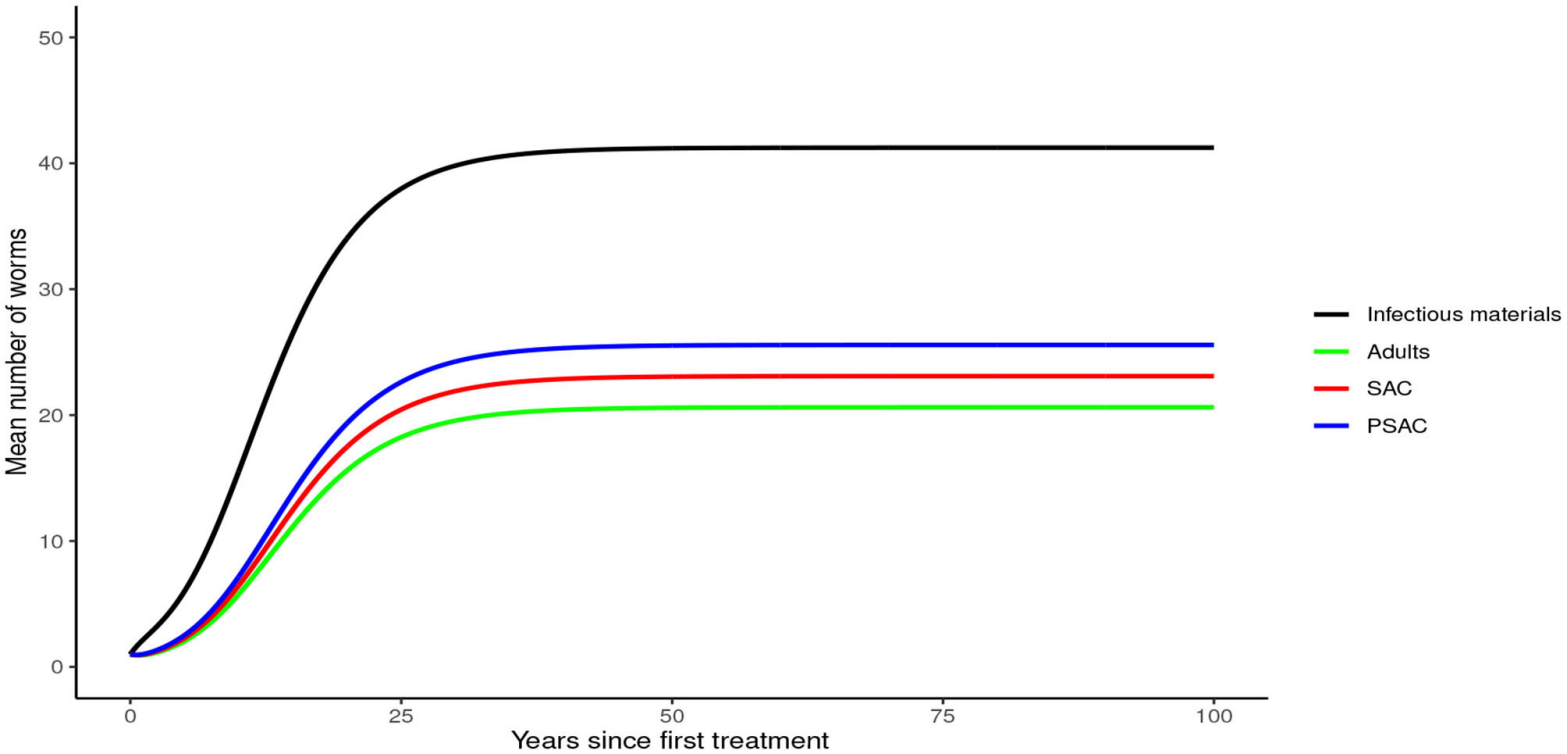
Model 1 solution for *Trichuris trichiura*: Here, no intervention was assumed.

In the absence of any intervention, *Trichuris trichiura* rose to an endemic equilibrium of between 18 to 25 mean worm burden among the hosts and 40 mean infectious materials in the environment. We note that it took a longer time for *T. trichiura* to achieve endemic equilibrium compared to the other two STH species (see Figure 10).

**Figure 11:**
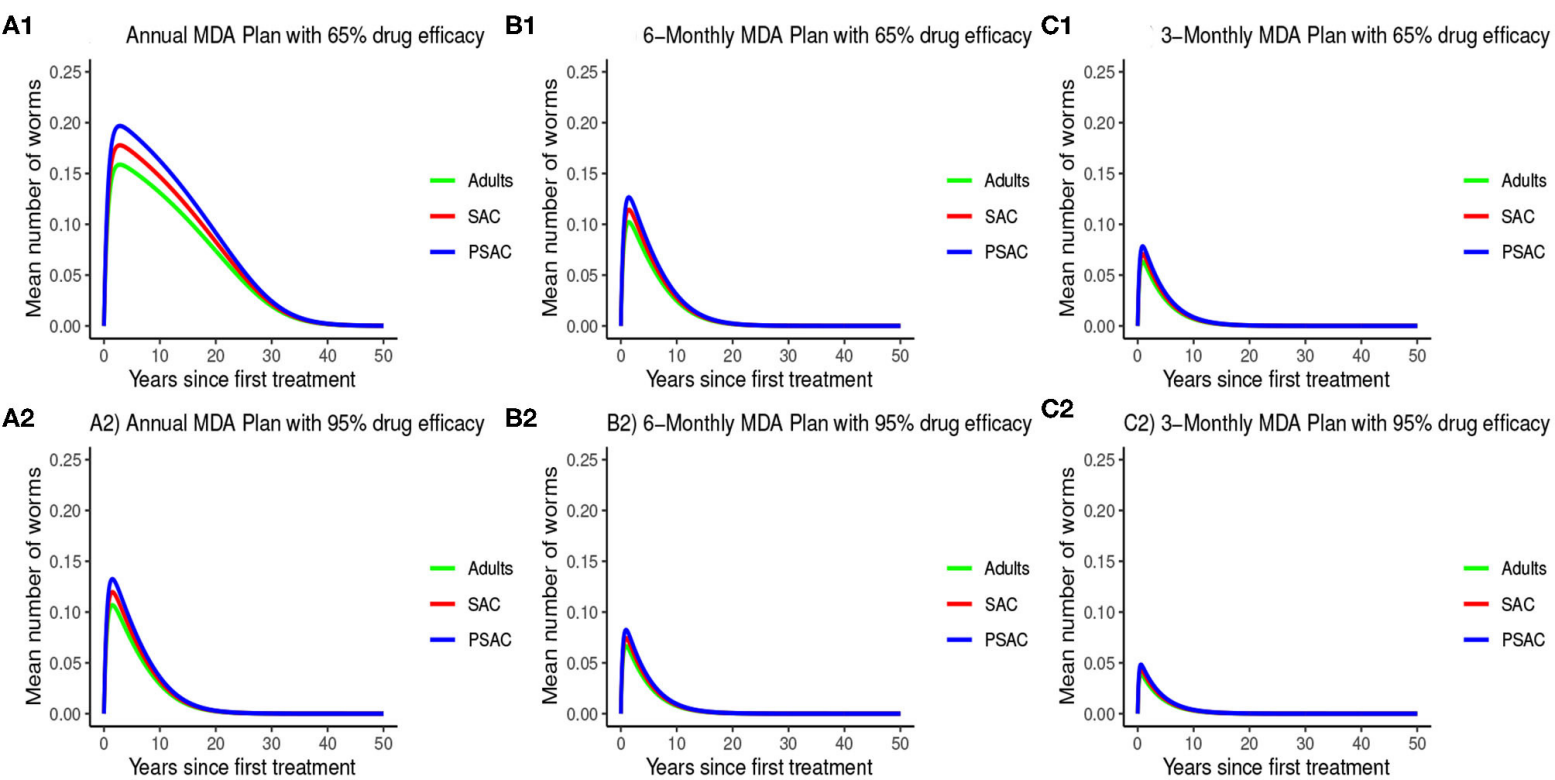
Model 2 solution for *Trichuris trichiura*: We assumed various MDA plans as indicated in **(A–C)** i.e., τ = 1.0 for yearly plan, τ = 0.5 for 6-monthly plan, and τ = 0.25 for 3-monthly plan. Additionally, for each MDA plan we varied the drug efficacy (h) as either 65% or 95%. We assumed treatment coverage of 75% for each host group. These assumptions followed the current WHO and NSBD guidelines (13).

The impact of MDA on *T. trichiura* for various MDA plans and drug efficacy is demonstrated in Figure 11. Clearly we see that it will take longer than 10 years to eliminate *T. trichiura* under any of the MDA plans compared to the other species. Specifically, for *T. trichiura* we compared various drug efficacy levels for each MDA plan, and we see that the higher the drug efficacy level the shorter the elimination period. Unlike the other species, we noted that MDA alone will not eliminate *T. trichiura* in the short period; though, it will substantially suppress the worm burden (Figure 11). It is important to see the influence of varying drug efficacy on *T. trichiura* since the current drug (albendazole) being used by the Kenyan program has been shown to be less efficacious against it (40). Hence there is need of a highly efficacious drug as is the case when drug combination is used (40).

**Figure 12:**
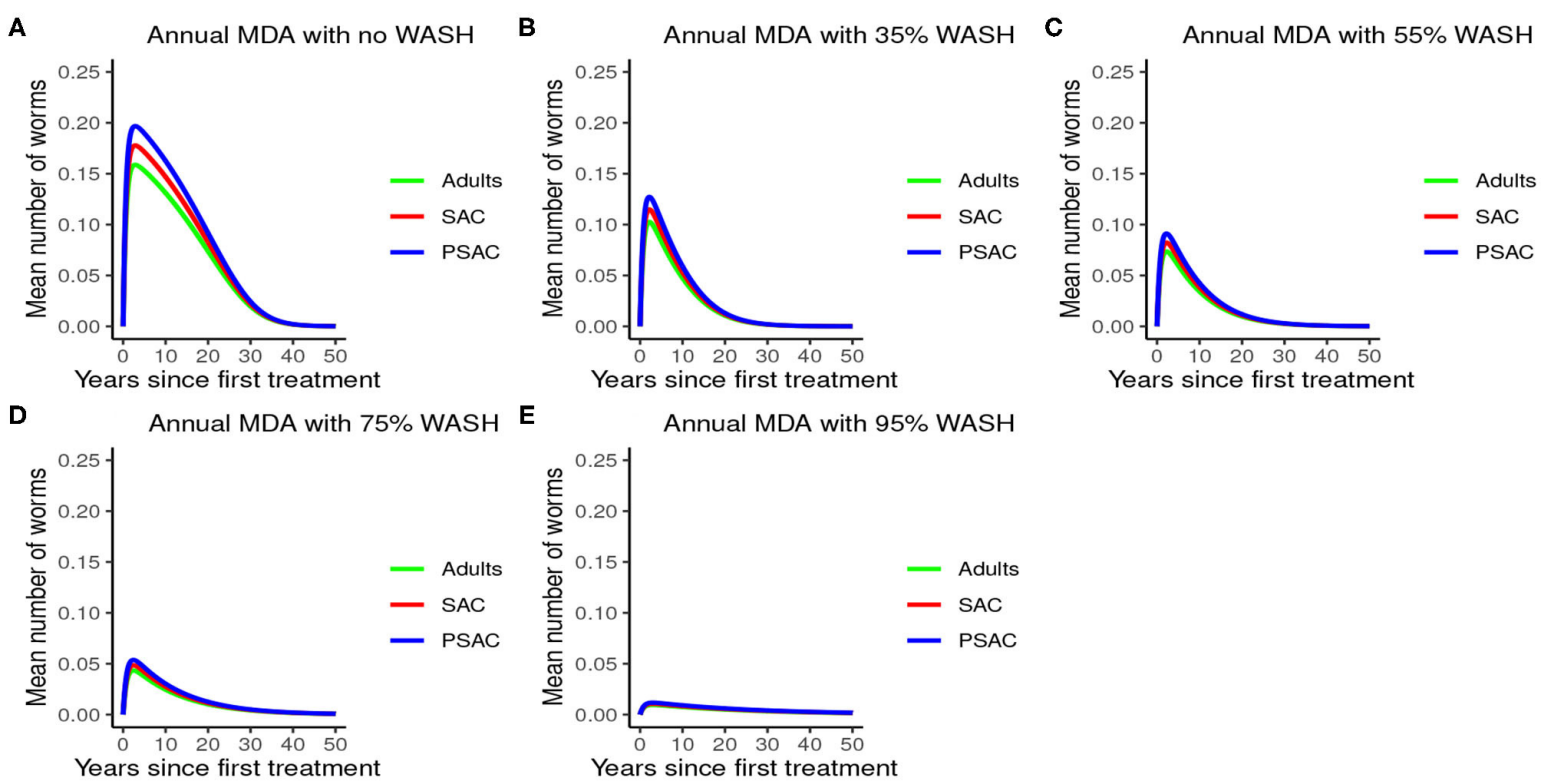
Model 3 solution for *Trichuris trichiura*: We assumed annual MDA plan with various WASH levels as indicated in **(A–E)** i.e., ϕ = 0 for no WASH, ϕ = 0.35 for 35% WASH, ϕ = 0.55 for 55% WASH, ϕ = 0.75 for 75% WASH, and ϕ = 0.95 for 95% WASH. Further, we assumed treatment coverage of 75% for each host group and drug efficacy (h) of 65%. These assumptions followed the current WHO and NSBD guidelines (13).

The impact of WASH on *T. trichiura* is well-illustrated in Figure 12. Just like with the other species, complementing annual MDA with WASH was shown to be very effective in reducing the elimination period and worm burden. We observed that the WASH intervention was very effective when administered at a very high coverage level of 95%.

#### 3.2.4. Sensitivity of *R*_0_ to MDA and WASH Interventions Parameters

**Figure 13:**
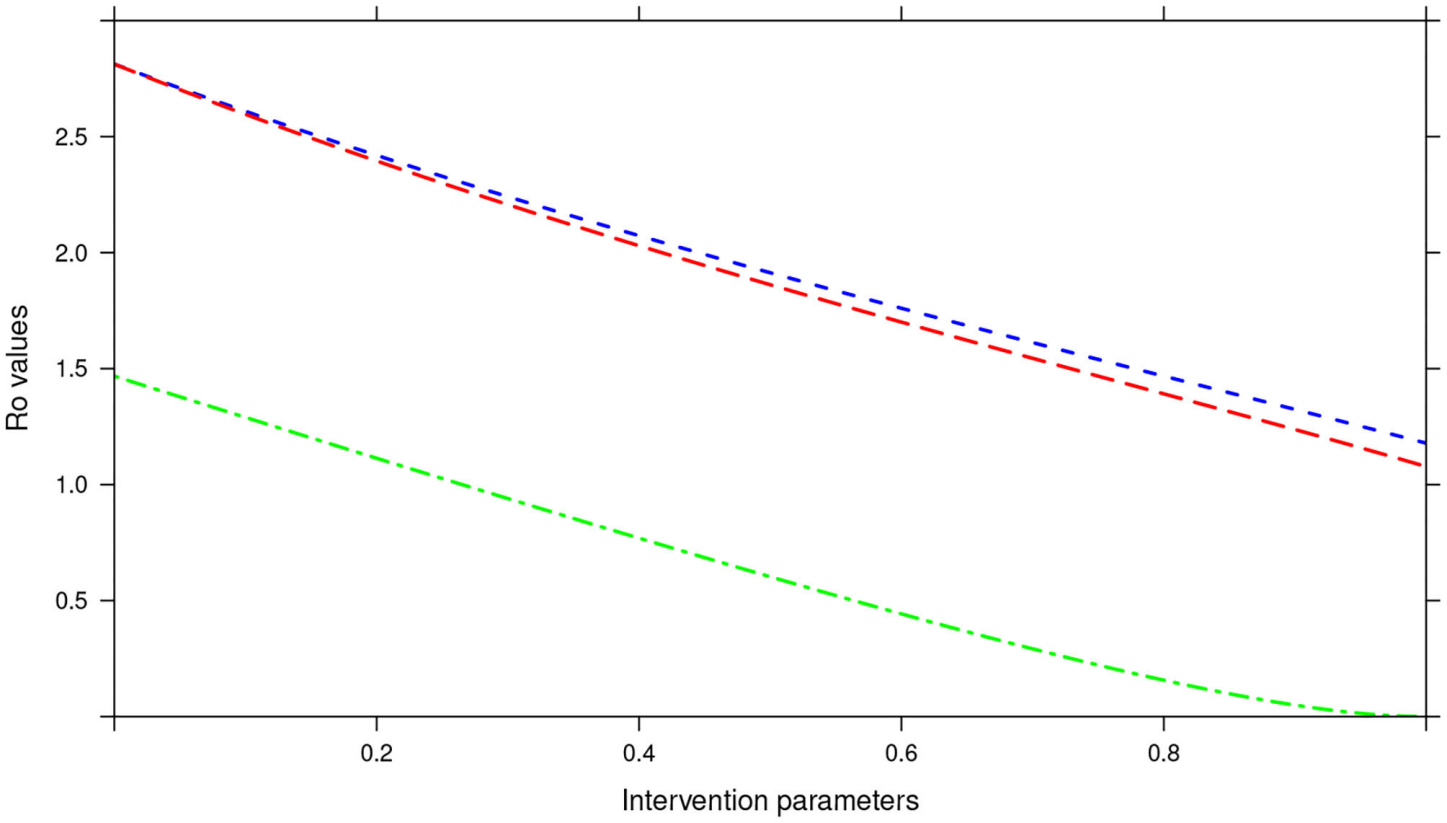
Showing the impact of MDA and WASH interventions parameters on *R*_0_ assuming an annual MDA plan. The parameters evaluated were drug efficacy (blue line), proportion of individuals treated (red line) and WASH coverage (green line). The values of all the parameters ranged from 0 to 100% (or 0 to 1.0).

During the assessment of the impact of key parameters associated with the MDA and WASH interventions, we observed that even if drug efficacy and proportion of individuals treated (treatment coverage) were increased to 100%, the annual MDA plan did not result in *R*_0_ being less than 1 (see Figure 13). This implied that annual MDA alone can not guarantee effective control or elimination of STH in the short period.

**Figure 14:**
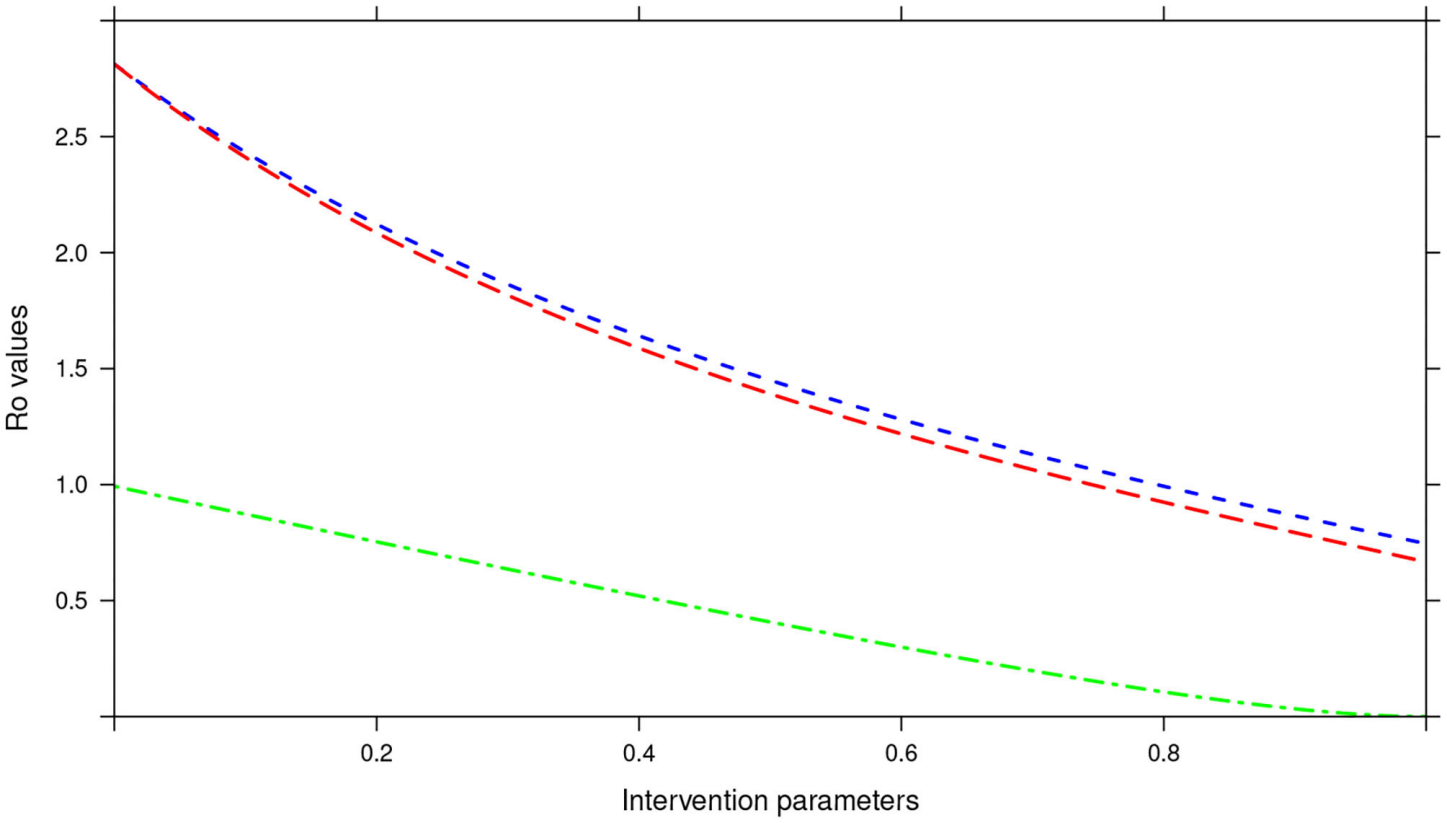
Showing the impact of MDA and WASH interventions parameters on *R*_0_ assuming a bi-annual MDA plan. The parameters evaluated were drug efficacy (blue line), proportion of individuals treated (red line) and WASH coverage (green line). The values of all the parameters ranged from 0 to 100% (or 0 to 1.0).

In the bi-annual MDA plan, increasing the two parameters (drug efficacy and treatment coverage) to 100% resulted in *R*_0_ < 1, precisely the final *R*_0_ value achieved was 0.67 (see Figure 14). With the final *R*_0_ value here being below 1, it implied effective control of the infections and possible elimination in the long run.

**Figure 15:**
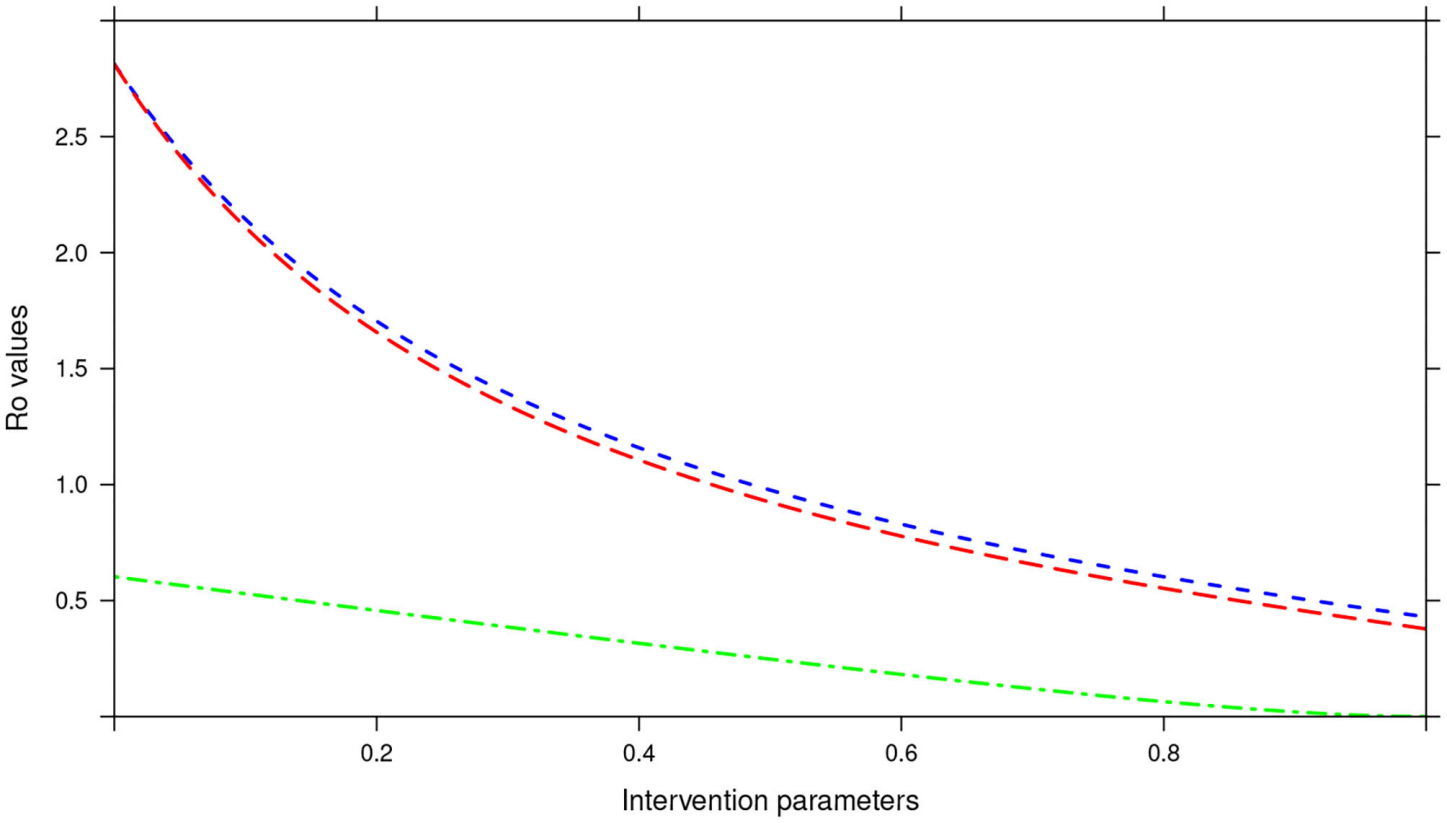
Showing the impact of MDA and WASH interventions parameters on *R*_0_ assuming a tri-annual MDA plan. The parameters evaluated were drug efficacy (blue line), proportion of individuals treated (red line) and WASH coverage (green line). The values of all the parameters ranged from 0 to 100% (or 0 to 1.0).

Further, in the tri-annual MDA plan, varying the two parameters (drug efficacy and treatment coverage) resulted in *R*_0_ < 1, precisely the final *R*_0_ value achieved was 0.38 (see Figure 15). Again implying effective control of the infections and possible elimination in the short run.

Importantly, we noted that adding WASH intervention to any of the three MDA plans and varying it to 100% resulted in the final *R*_0_ = 0 (see Figures 13–15). This implied immediate impact of WASH that would accelerate attainment of complete elimination of the infections in the short run.

Impact of the treatment intervals (rounds) on *R*_0_ was demonstrated in Supplementary Figure 1. The results indicated that varying the treatment intervals to shorter periods (or rather higher number of rounds), resulted in *R*_0_ values being gradually reduced up to zero.

#### 3.2.5. Impact of Interventions on Infectious Materials in the Environment

The model further assessed the impact of the interventions (MDA and WASH) on the mean number of infectious materials in the environment as shown in Supplementary Figure 2. From the analysis, we observed that MDA (treatment offered to host) was more effective in reducing the elimination period of the infectious materials in the environment compared to WASH (primarily prevention of infectious materials acquisition by the host). The elimination period reduced as the MDA rounds increased.

## 4. Discussion

In this study, we presented the first STH transmission elimination mathematical model based on the STH dynamics specific to Kenya. The study used deterministic model to assess the impact of key interventions (MDA and WASH) on the mean worm burden and elimination period. These results are important to the STH control program in Kenya since it clearly demonstrated the impact of the interventions as well as the projected elimination period of the infections in the country. The key novelty in this study is the illustrated impact among all the three common species of STH, a rare feature in most of the previous STH transmission models (11, 16, 24, 28). This is an important aspect in our modeling since most control programs are now focusing on species-specific interventions (41, 42).

The model analyses showed that the reduction of worm burden and elimination period depended heavily on four parameter groups; drug efficacy (*h*), treatment rounds (τ), proportion of individuals reached with treatment (*g*), and WASH coverage (ϕ). Parameters τ, *h*, and *g* defined properties of the MDA intervention and ϕ properties of the WASH intervention. The analyses suggested that the impact of the interventions were very sensitive to the changes in values of the four parameter groupings, similar observation was made by Truscott and colleagues (26). Together all these parameters affected the *R*_0_, which is a "summary" parameter for the intensity of the transmission (43). Varying these parameters to higher values reduced *R*_0_ to low levels (below 1). Even though previous studies have estimated *R*_0_ for different STH species using variety of methods (28, 38), we focused its estimation to the STH transmission dynamics in Kenya.

From these analyses, we found that the endemic mean worm burdens in the absence of any interventions were low especially for hookworm and *T. trichiura*, and ranged from 140 to 180 for the case of *A. lumbricoides*, 20 to 35 for hookworm and 18 to 25 for *T. trichiura*. Previous studies have reported similar ranges of endemic worm burdens (16, 25–27, 31, 32, 44). However, this was still significantly higher than the critical levels below which a worm finding a mate becomes a problem [defined as critical worm burden, *M*_*SR*_, with approximate cut-off point of about one worm per host (26)]. If worm burdens goes below *M*_*SR*_, the decline in egg production reaches a point at which it balances the ability of the worms and infectious materials to persist in the host or environment, illustrating a "breakpoint" (43). Below the breakpoint are very low values of worm burden and characterized by stable parasite-free state (45). All our evaluated interventions, when administered at an appropriate level, had the effect of significantly reducing the worm burdens in all the three age groups to below *M*_*SR*_. Hence, this is viewed to have a negative impact on parasite production and subsequent transmission potential since there would be much fewer instances of both sexes infecting the same host. In context of this work, the annual MDA alone with drug efficacy of 80%, achieved *M*_*SR*_ after about 15 rounds of annual treatment. The 3-monthly MDA achieved it after about 5 years. The results presented here are comparable to other STH modeling studies (16, 26, 28).

An important advantage of our work is the demonstrated impact of WASH as a complementary intervention to an MDA program. Only few modeling studies have compared the impact of these two interventions in a single modeling study (46). The analyses showed that with annual MDA alone (see Figures 5, 8, 11), STH control programs would take very long to reach elimination. Actually, during the assessment of the sensitivity of *R*_0_ to the intervention parameters, we showed that annual MDA programs would not drive *R*_0_ to below 1 (suggesting resurgence of infections after the program implementation) (see Figure 13). However, if the annual program is complemented with WASH at a sufficiently higher coverage level, preferably 95%, then the elimination period would be reached within a shorter period (see Figures 6, 9, 12). This is supported by the fact that whenever WASH parameter was added to the models and varied to maximum levels, *R*_0_ reduced to zero, implying complete elimination (see Figures 13–15). These analyses results offer the STH control programs the flexibility to choose on whether to continue with MDA alone (and at what level of treatment rounds) or to complement it with WASH.

Additional advantage of this work is the prediction of the lifetime of the infectious materials in the environment in relation to the two interventions as presented in Supplementary Figure 2. Majority of the previous STH models ignored the dynamics of the infectious materials in the environment. However, the few studies that incorporated it assumed that the infectious materials are highly infectious and followed the parasite dynamics in the host. In essence, this assumption is true when the influence of sexual reproduction on parasite population is considered in the absence of a regular intervention program (for example periodic treatment) (26). However, in the presence of a regular periodic treatment of a particular age group, the environment (reservoir) is viewed as a source of new infections and the larger the lifespan of the infectious materials, the more the environment becomes potentially infectious (47). Our results demonstrated that regular periodic treatment of the hosts (through MDA) was more effective in reducing the period of the infectiousness of the environment. However, we observed that WASH intervention did not offer significant advantage in reducing that period (see Supplementary Figure 2E). This is mainly true because treatment would “kill” the adult worms in the host hence reducing the host's ability to contaminate the environment.

### 4.1. Study Limitations

This study was not without limitations. (1) As much as we tried to estimate our parameters from field studies conducted in Kenya, there were far less large-scale studies conducted separately targeting each of the three age groups. Majority of the studies were on school-aged children. This forced us to estimate some of the parameter values for certain age groups or refer to previous model parameters. If future infection transmission models in Kenya are to be improved, investment in field surveys on all the three age groups to measure parameter combinations is desired. (2) Even though the Kenyan deworming program measured, through field surveys, outcomes (mean number of eggs and prevalence) slightly different from those we modeled (mean worm burden and elimination period), this variation did not cause any bias to the results or the overall study aim. All these outcomes helped define the infection control and elimination. (3) This current study did not evaluate the spatial heterogeneity, interaction terms between the parasites, possibility of zoonotic infections being observed in our study population, or applicability of our model to different regions of Kenya. Even though these considerations would be important since the control efforts are focused particularly towards transmission interruption, they were out of scope of the current analysis. Some of these limitations would be addressed in our future modeling analyses.

## 5. Conclusion

Kenya launched its breaking transmission strategy (BTS) in 2019, and therefore these results would be timely in guiding the implementation of this strategy. Specifically, the models demonstrated that for infections to be eliminated using MDA alone in a short time period, 3-monthly MDA plan is desired. However, complementation of annual MDA with WASH at a desired coverage level was most effective.

## Data Availability Statement

The original contributions presented in the study are included in the article/Supplementary Material, further inquiries can be directed to the corresponding author/s.

## Ethics Statement

The studies involving human participants were reviewed and approved by Kenya Medical Research Institute (KEMRI)'s Scientific and Ethics Review Unit (SSC Number 2206). Written informed consent to participate in this study was provided by the participants' legal guardian/next of kin.

## Author Contributions

CO conceptualized the study, formulated the model, developed the R codes and analyzed the models, and wrote the draft manuscript. CM provided the field data, interpreted the parasitological results, and reviewed the draft manuscript. GM and NO conceptualized the study, formulated the model, reviewed the draft manuscript and provided overall scientific guidance. All authors participated in the interpretation of the findings, read, and approved the final manuscript.

## Conflict of Interest

The authors declare that the research was conducted in the absence of any commercial or financial relationships that could be construed as a potential conflict of interest.

## References

[1] Pullan RL Smith JL Jasrasaria R Brooker SJ. Global numbers of infection and disease burden of soil transmitted helminth infections in 2010. Parasit Vectors. (2014) 7:37. 10.1186/1756-3305-7-37 24447578 PMC3905661

[2] WHO. Accelerating Work to Overcome the Global Impact of Neglected Tropical Diseases: A Roadmap for Implementation: Executive Summary. World Health Organization (2012).

[3] WHO. Investing to Overcome the Global Impact of Neglected Tropical Diseases: Third WHO Report on Neglected Tropical Diseases 2015. Vol. 3. World Health Organization (2015).

[4] Knopp S Steinmann P Keiser J Utzinger J. Nematode infections: soil-transmitted helminths and Trichinella. Infect Dis Clin. (2012) 26:341–58. 10.1016/j.idc.2012.02.006 22632643

[5] Coffeng LE Vaz Nery S Gray DJ Bakker R de Vlas SJ Clements AC. Predicted short and long-term impact of deworming and water, hygiene, and sanitation on transmission of soil-transmitted helminths. PLoS Negl Trop Dis. (2018) 12:e0006758. 10.1371/journal.pntd.0006758 30522129 PMC6283645

[6] Freeman MC Garn JV Sclar GD Boisson S Medlicott K Alexander KT. The impact of sanitation on infectious disease and nutritional status: a systematic review and meta-analysis. Int J Hyg Environ Health. (2017) 220:928–49. 10.1016/j.ijheh.2017.05.007 28602619

[7] WHO. Working to Overcome the Global Impact of Neglected Tropical Diseases: First WHO Report on Neglected Tropical Diseases. World Health Organization (2010).

[8] Keiser J Utzinger J. The drugs we have and the drugs we need against major helminth infections. Adv Parasitol. (2010) 73:197–230. 10.1016/S0065-308X(10)73008-6 20627144

[9] Muchiri EM Thiong'o FW Magnussen P Ouma JH. A comparative study of different albendazole and mebendazole regimens for the treatment of intestinal infections in school children of Usigu Division, western Kenya. J Parasitol. (2001) 87:413–8. 10.1645/0022-3395(2001)087[0413:ACSODA]2.0.CO;2 11318574

[10] WHO. Guideline: Preventive Chemotherapy to Control Soil-Transmitted Helminth Infections in At-Risk Population Groups. World Health Organization (2017). 29578660

[11] Anderson R Farrell S Turner H Walson J Donnelly CA Truscott J. Assessing the interruption of the transmission of human helminths with mass drug administration alone: optimizing the design of cluster randomized trials. Parasit Vectors. (2017) 10:93. 10.1186/s13071-017-1979-x 28212667 PMC5316156

[12] Ásbjörnsdóttir KH Ajjampur SSR Anderson RM Bailey R Gardiner I Halliday KE. Assessing the feasibility of interrupting the transmission of soil-transmitted helminths through mass drug administration: the DeWorm3 cluster randomized trial protocol. PLoS Negl Trop Dis. (2018) 12:e0006166. 10.1371/journal.pntd.0006253 29346377 PMC5773085

[13] WHO. Helminth Control in School-Age Children: A Guide for Managers of Control Programmes. World Health Organization (2011).

[14] Speich B Moser W Ali SM Ame SM Albonico M Hattendorf J. Efficacy and reinfection with soil-transmitted helminths 18-weeks post-treatment with albendazole-ivermectin, albendazole-mebendazole, albendazole-oxantel pamoate and mebendazole. Parasit Vectors. (2016) 9:123. 10.1186/s13071-016-1406-8 26935065 PMC4776366

[15] Farrell SH Truscott JE Anderson RM. The importance of patient compliance in repeated rounds of mass drug administration (MDA) for the elimination of intestinal helminth transmission. Parasit Vectors. (2017) 10:1–12. 10.1186/s13071-017-2206-5 28606164 PMC5469187

[16] Chan M Guyatt H Bundy DA Medley G. The development and validation of an age-structured model for the evaluation of disease control strategies for intestinal helminths. Parasitology. (1994) 109:389–96. 10.1017/S0031182000078422 7970893

[17] Miguel E Kremer M. Worms: identifying impacts on education and health in the presence of treatment externalities. Econometrica. (2004) 72:159–217. 10.1111/j.1468-0262.2004.00481.x

[18] Hicks JH Kremer M Miguel E. The case for mass treatment of intestinal helminths in endemic areas. PLoS Negl Trop Dis. (2015) 9:e0004214. 10.1371/journal.pntd.0004214 26492528 PMC4619642

[19] Mwandawiro CS Nikolay B Kihara JH Ozier O Mukoko DA Mwanje MT. Monitoring and evaluating the impact of national school-based deworming in Kenya: study design and baseline results. Parasit Vectors. (2013) 6:198. 10.1186/1756-3305-6-198 23829767 PMC3723516

[20] Mwandawiro C Okoyo C Kihara J Simiyu E Kepha S Campbell SJ. Results of a national school-based deworming programme on soil-transmitted helminths infections and schistosomiasis in Kenya: 2012–2017. Parasit Vectors. (2019) 12:76. 10.1186/s13071-019-3322-1 30732642 PMC6367841

[21] Okoyo C Birgit N Kihara J Simiyu E Garn JV Freeman MC. Monitoring the impact of a national school based deworming programme on soil-transmitted helminths in Kenya: the first three years, 2012–2014. Parasit Vectors. (2016) 9:408. 10.1186/s13071-016-1679-y 27457129 PMC4960809

[22] Okoyo C Campbell SJ Williams K Simiyu E Owaga C Mwandawiro C. Prevalence, intensity and associated risk factors of soil-transmitted helminth and schistosome infections in Kenya: impact assessment after five rounds of mass drug administration in Kenya. PLoS Negl Trop Dis. (2020) 14:e0008604. 10.1371/journal.pntd.0008604 33027264 PMC7540847

[23] Giardina F Coffeng LE Farrell SH Vegvari C Werkman M Truscott JE. Sampling strategies for monitoring and evaluation of morbidity targets for soil-transmitted helminths. PLoS Negl Trop Dis. (2019) 13:e0007514. 10.1371/journal.pntd.0007514 31242194 PMC6615707

[24] Medley G Guyatt H Bundy D. A quantitative framework for evaluating the effect of community treatment on the morbidity due to ascariasis. Parasitology. (1993) 106:211–21. 10.1017/S0031182000075016 8446474

[25] Chan M Bradley M Bundy D. Transmission patterns and the epidemiology of hookworm infection. Int J Epidemiol. (1997) 26:1392–400. 10.1093/ije/26.6.1392 9447422

[26] Truscott J Hollingsworth TD Anderson R. Modeling the interruption of the transmission of soil-transmitted helminths by repeated mass chemotherapy of school-age children. PLoS Negl Trop Dis. (2014) 8:e3323. 10.1371/journal.pntd.0003323 25474477 PMC4256169

[27] Truscott JE Hollingsworth TD Brooker SJ Anderson RM. Can chemotherapy alone eliminate the transmission of soil transmitted helminths? Parasit Vectors. (2014) 7:266. 10.1186/1756-3305-7-266 24916278 PMC4079919

[28] Truscott J Turner H Farrell S Anderson R. Soil-transmitted helminths: mathematical models of transmission, the impact of mass drug administration and transmission elimination criteria. Adv Parasitol. (2016) 94:133–98. 10.1016/bs.apar.2016.08.002 27756454

[29] Anderson RM May RM. Population dynamics of human helminth infections: control by chemotherapy. Nature. (1982) 297:557. 10.1038/297557a0 7088139

[30] Anderson RM May RM. Vaccination and herd immunity to infectious diseases. Nature. (1985) 318:323–9. 3906406 10.1038/318323a0

[31] Anderson RM May RM. Infectious Diseases of Humans: Dynamics and Control. Oxford: Oxford University Press (1992).

[32] Anderson RM Truscott JE Pullan RL Brooker SJ Hollingsworth TD. How effective is school-based deworming for the community-wide control of soil-transmitted helminths? PLoS Negl Trop Dis. (2013) 7:e2027. 10.1371/journal.pntd.0002027 23469293 PMC3585037

[33] WHO. 2030 Targets for Soil-Transmitted Helminthiases Control Programmes. World Health Organization (2020).

[34] Ndayishimiye O Ortu G Magalhaes RJS Clements A Willems J Whitton J. Control of neglected tropical diseases in Burundi: partnerships, achievements, challenges, and lessons learned after four years of programme implementation. PLoS Negl Trop Dis. (2014) 8:e2684. 10.1371/journal.pntd.0002684 24785993 PMC4006741

[35] World Health Organization. A Master Plan for National Neglected Tropical Diseases Programmes in the African Region. (2012).

[36] Albonico M Allen H Chitsulo L Engels D Gabrielli AF Savioli L. Controlling soil-transmitted helminthiasis in pre-school-age children through preventive chemotherapy. PLoS Negl Trop Dis. (2008) 2:e126. 10.1371/journal.pntd.0000126 18365031 PMC2274864

[37] Roberts M Andreasen V Lloyd A Pellis L. Nine challenges for deterministic epidemic models. Epidemics. (2015) 10:49–53. 10.1016/j.epidem.2014.09.006 25843383 PMC4996659

[38] Lambura AG Mwanga GG Luboobi L Kuznetsov D. Mathematical model for optimal control of soil-transmitted helminth infection. Comput Math Methods Med. (2020) 2020:6721919. 10.1155/2020/6721919 32802152 PMC7416292

[39] KNBS. Kenya Census 2019. Nairobi: Government Press (2019).

[40] Vercruysse J Behnke JM Albonico M Ame SM Angebault C Bethony JM. Assessment of the anthelmintic efficacy of albendazole in school children in seven countries where soil-transmitted helminths are endemic. PLoS Negl Trop Dis. (2011) 5:e948. 10.1371/journal.pntd.0000948 21468309 PMC3066140

[41] Knopp S Mohammed KA Rollinson D Stothard JR Khamis IS Utzinger J. Changing patterns of soil-transmitted helminthiases in Zanzibar in the context of national helminth control programs. Am J Trop Med Hyg. (2009) 81:1071–8. 10.4269/ajtmh.2009.09-0377 19996439

[42] WHO. Framework for Control and Prevention of Soil-Transmitted Helminthiases in the WHO European Region 2016–2020. (2016).

[43] Basáñez MG McCarthy JS French MD Yang GJ Walker M Gambhir M. A research agenda for helminth diseases of humans: modelling for control and elimination. PLoS Negl Trop Dis. (2012) 6:e1548. 10.1371/journal.pntd.0001548 22545162 PMC3335861

[44] Isham V. Stochastic models of host-macroparasite interaction. Ann Appl Probabil. (1995) 5:720–40.

[45] Hardwick RJ Vegvari C Truscott JE Anderson RM. The breakpoint of soil-transmitted helminths with infected human migration. J. Theor. Biol. (2020) 486:110076. 10.1016/j.jtbi.2019.110076 31733259 PMC6977101

[46] Mekete K Ower A Dunn J Sime H Tadesse G Abate E. The Geshiyaro Project: a study protocol for developing a scalable model of interventions for moving towards the interruption of the transmission of soil-transmitted helminths and schistosome infections in the Wolaita zone of Ethiopia. Parasit Vectors. (2019) 12:1–12. 10.1186/s13071-019-3757-4 31665080 PMC6820996

[47] Park M Laksono B Sadler R Clements A Stewart DE. Household latrines to control environmental contamination and helminthiasis: an exploratory study in Indonesia. Int J Soc Sci Hum. (2015) 5:429. 10.7763/IJSSH.2015.V5.494

