# Supplementary material for: Modeling the Interruption of the Transmission of Soil-Transmitted Helminths Infections in Kenya: Modeling Deworming, Water, and Sanitation Impacts
Source: Front Public Health. 2021 Mar 24;9:637866. doi: 10.3389/fpubh.2021.637866 (PMC8024473; doi:10.3389/fpubh.2021.637866)
Supplement: Supplementary file 1 [file Data_Sheet_1.docx]

**Supplementary figures**


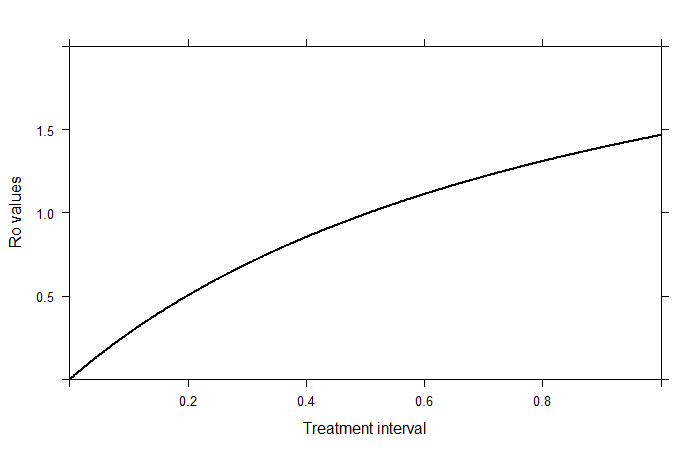


Figure S1: Showing the impact of treatment interval on $R_{o}$. The values of the treatment interval ranged from 0 to 1.0. For example, 1.0 indicated annual MDA, 0.5 bi-annual MDA, 0.25 tri-annual MDA, and 0 continuous treatment.


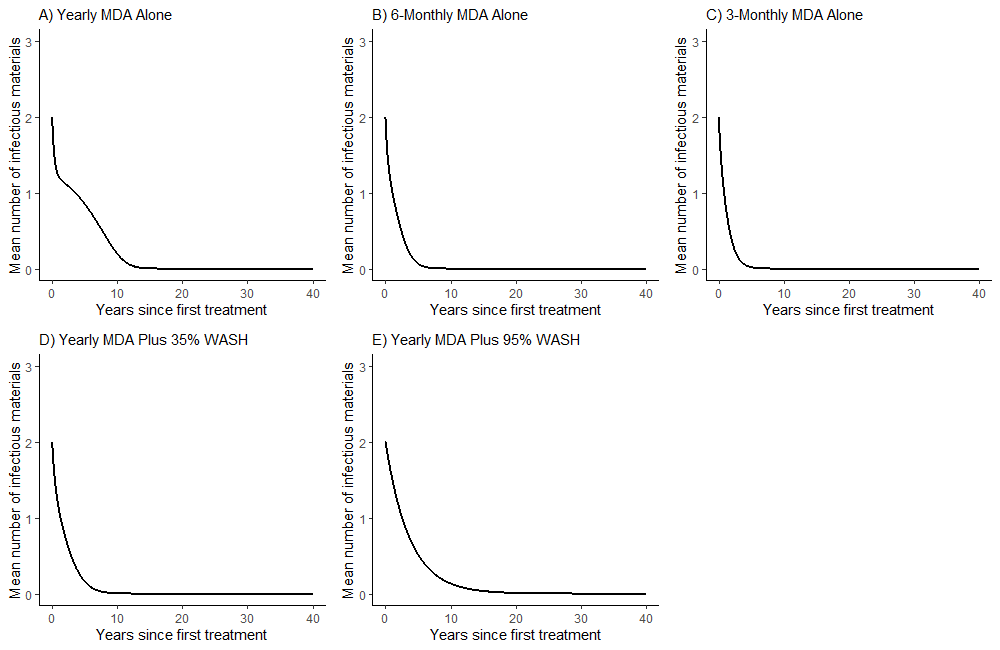


Figure S2: Showing the impact of the interventions (MDA and WASH) on the infectious materials in the environment (typical example of *Ascaris lumbricoides*). We assumed various MDA plans as indicated in panels (A) to (C) and WASH coverage as in panels (D) and (E). Additionally, we assumed treatment coverage (g) of 75% for each host group and drug efficacy (h) of 80%. These assumptions followed the current WHO and NSBD guidelines [13].
